# Supplementary figures and images for: Shotgun metagenomics captures more microbial diversity than targeted 16S rRNA gene sequencing for field specimens and preserved museum specimens
Source: PLoS One. 2023 Sep 19;18(9):e0291540. doi: 10.1371/journal.pone.0291540 (PMC10508626; doi:10.1371/journal.pone.0291540)

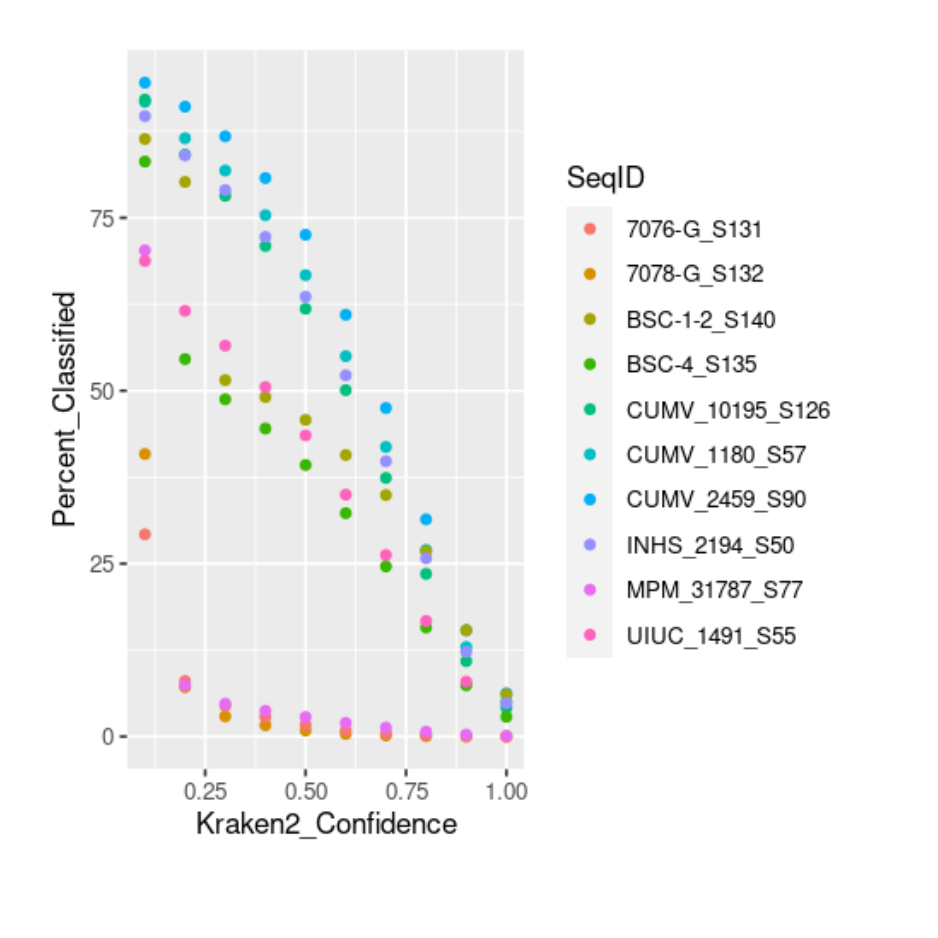

Supplement: S1 Fig — These data indicate a qualitative leveling-off at 0.8, which was subsequently used as a conservative confidence threshold (high confidence) so as to avoid incorrect classification of real microbial sequences as background data. (TIF) [file pone.0291540.s001.tif]

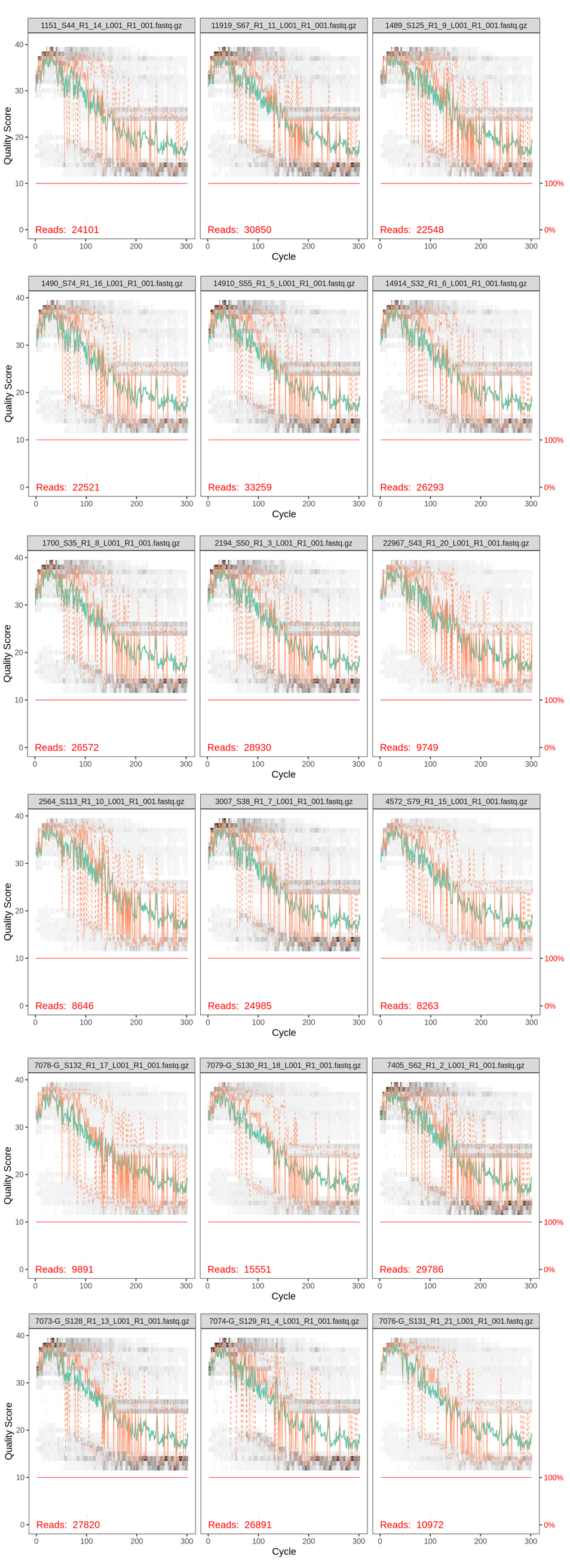

Supplement: S2 Fig — Quality score is given on the Y-axis and cycle number corresponding to read length is given on the X-axis. Reads were trimmed to 100bp based on these profiles, due to the quality decline for >100bp. (TIF) [file pone.0291540.s002.tif]

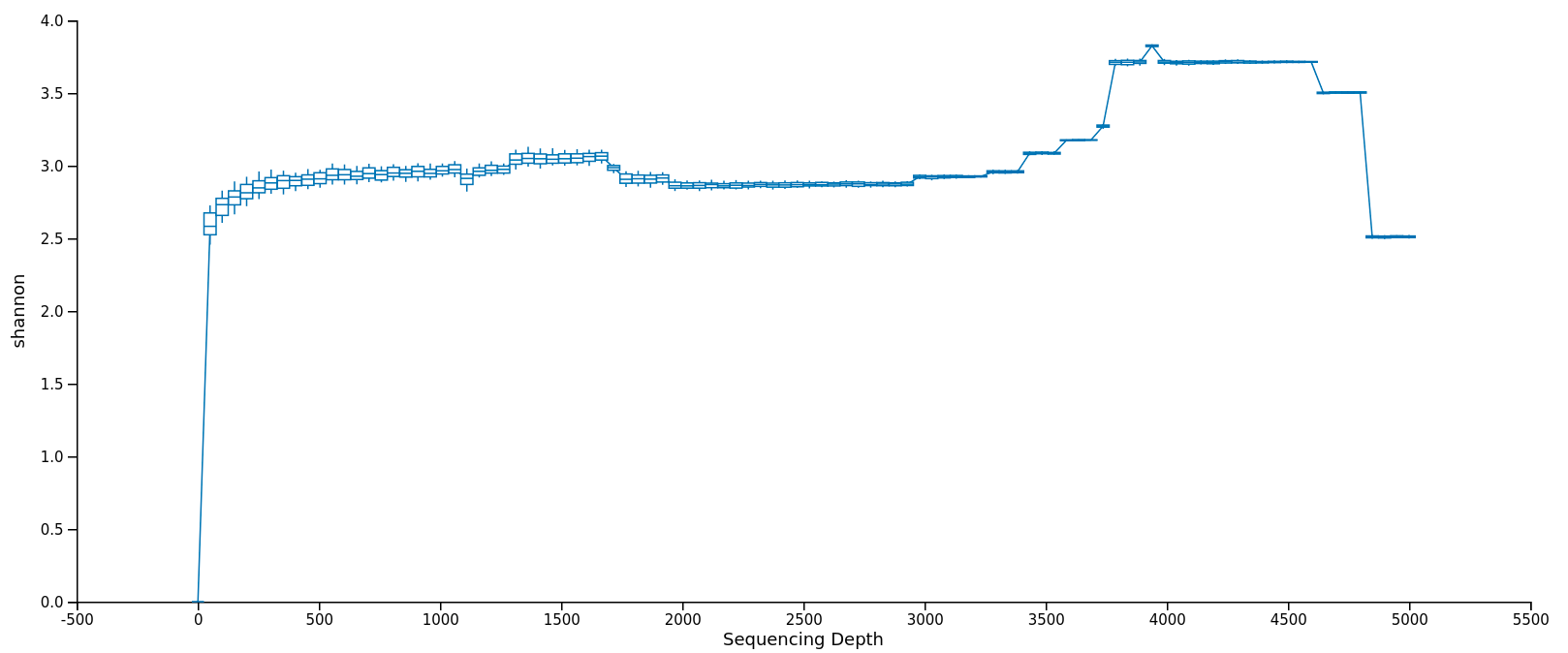

Supplement: S3 Fig — The lower and upper whiskers of the box plot are the 9th and 91st percentiles of the distribution (respectively), while the lower and upper extents of the box are the 25th and 75th percentiles of the distribution (respectively). The horizontal bar through the middle of the box is the median of the distribution (i.e., the 50th percentile). Outlier points of these distributions are not shown. The line chart connects the median Shannon diversity value distribution across the sampling depths. If a sampling depth is higher than the number of sequences in a sample, that sample is not included in the rarefaction plot at that sampling depth. (TIF) [file pone.0291540.s003.tif]

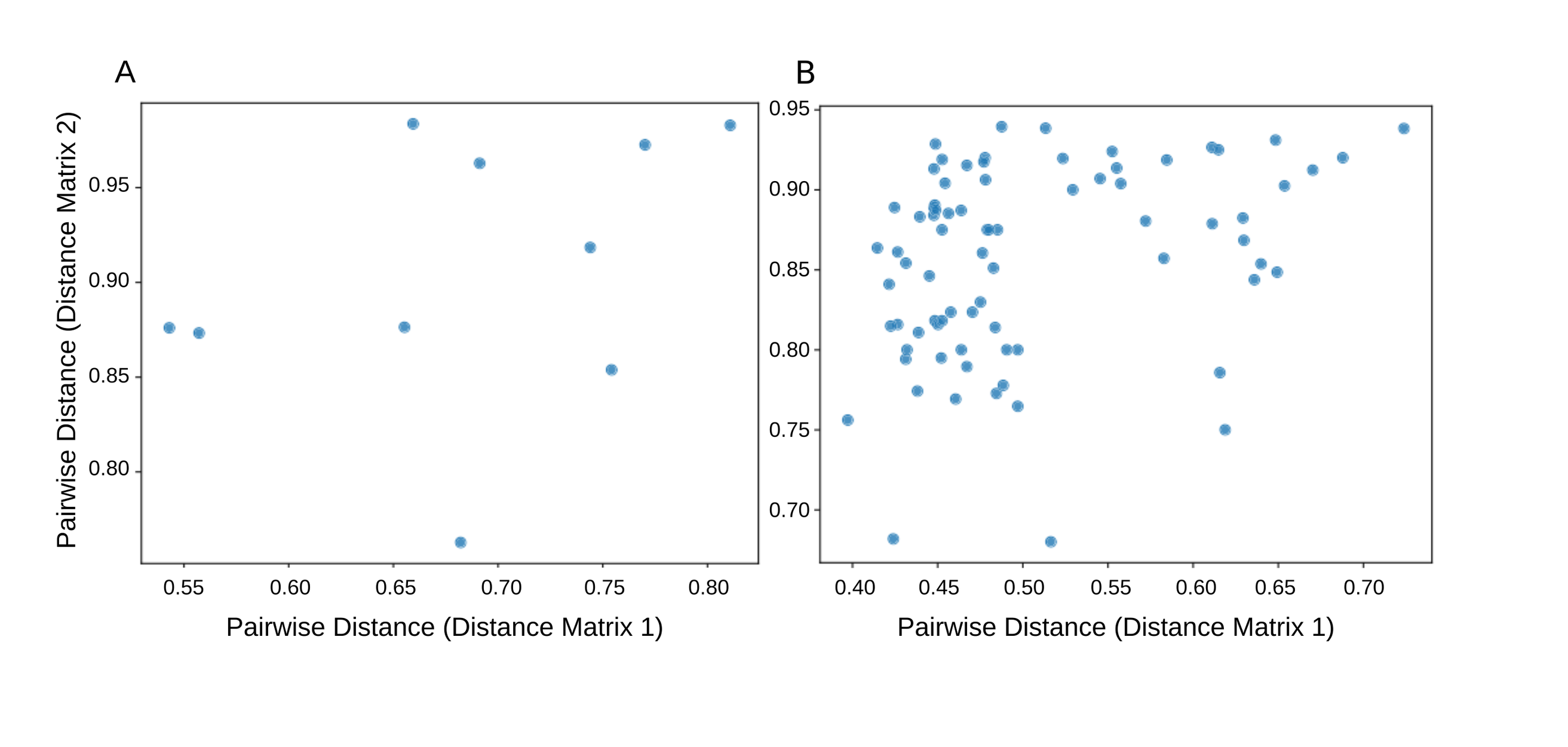

Supplement: S4 Fig — A–B. Mantel tests of Jaccard distance matrices from shotgun metagenomic sequencing data (Rep200 databases) and 16S rRNA gene sequencing data (Greengenes database). Fresh specimens are compared in Panel A and museum specimens are compared in panel B. (A) Spearman’s rho = 0.358, p-value = 0.451. (B) Spearman’s rho = 0.314, P-value = 0.158. (TIF) [file pone.0291540.s004.tif]

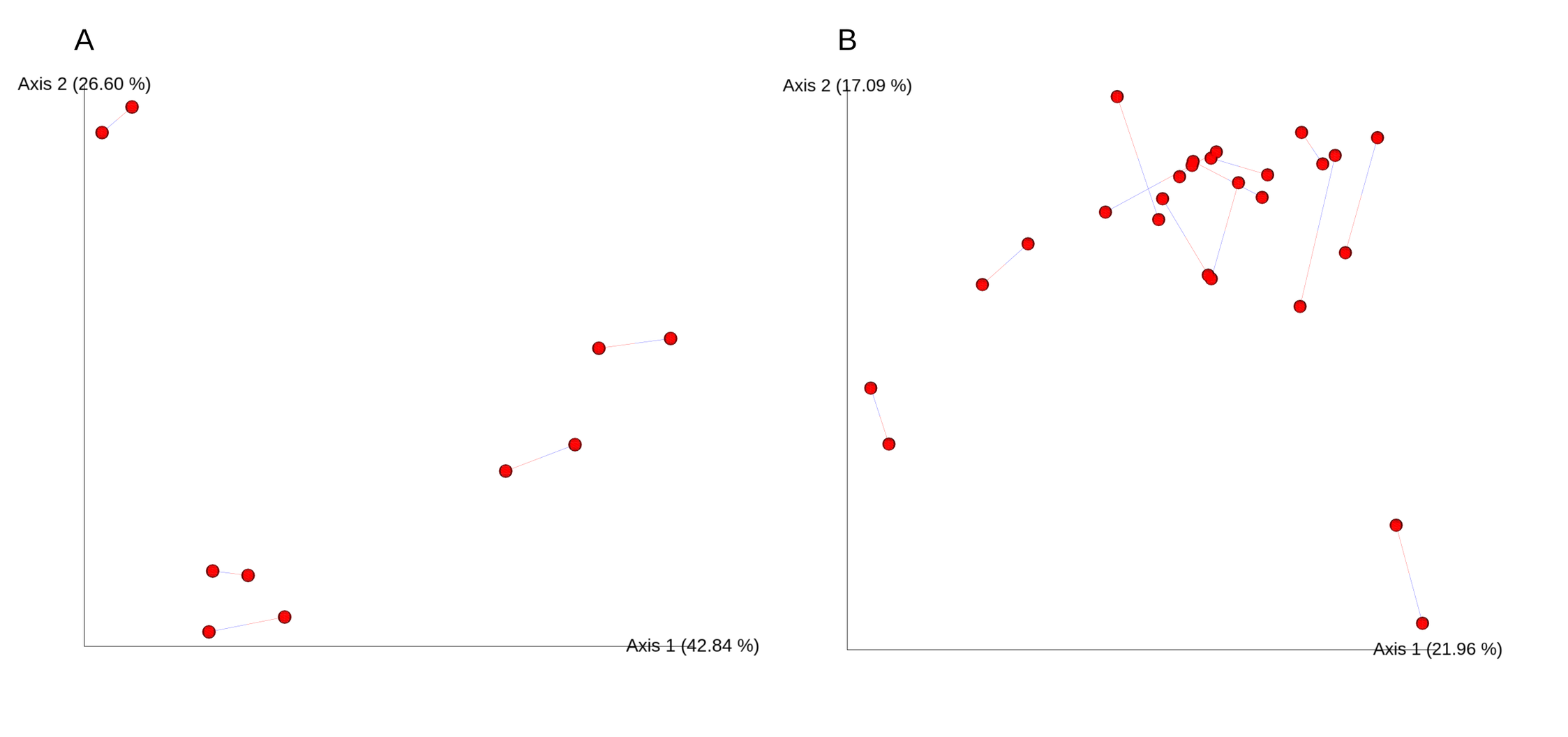

Supplement: S5 Fig — A–B. Procrustes analysis with Jaccard distance-matrix derived principal coordinates. The comparisons given are between: (A) Fresh specimen shotgun metagenomic sequencing (Rep200 database) and 16S rRNA gene sequencing (Greengenes database) with a true M2 = 0.0482; p-value (of true M2) = 0.219. (B) Museum-derived shotgun metagenomic sequencing (Rep200 database) and 16S rRNA gene sequencing (Greengenes database) with a true M2 = 0.481; p-value (of true M2) = 0.007. (TIF) [file pone.0291540.s005.tif]

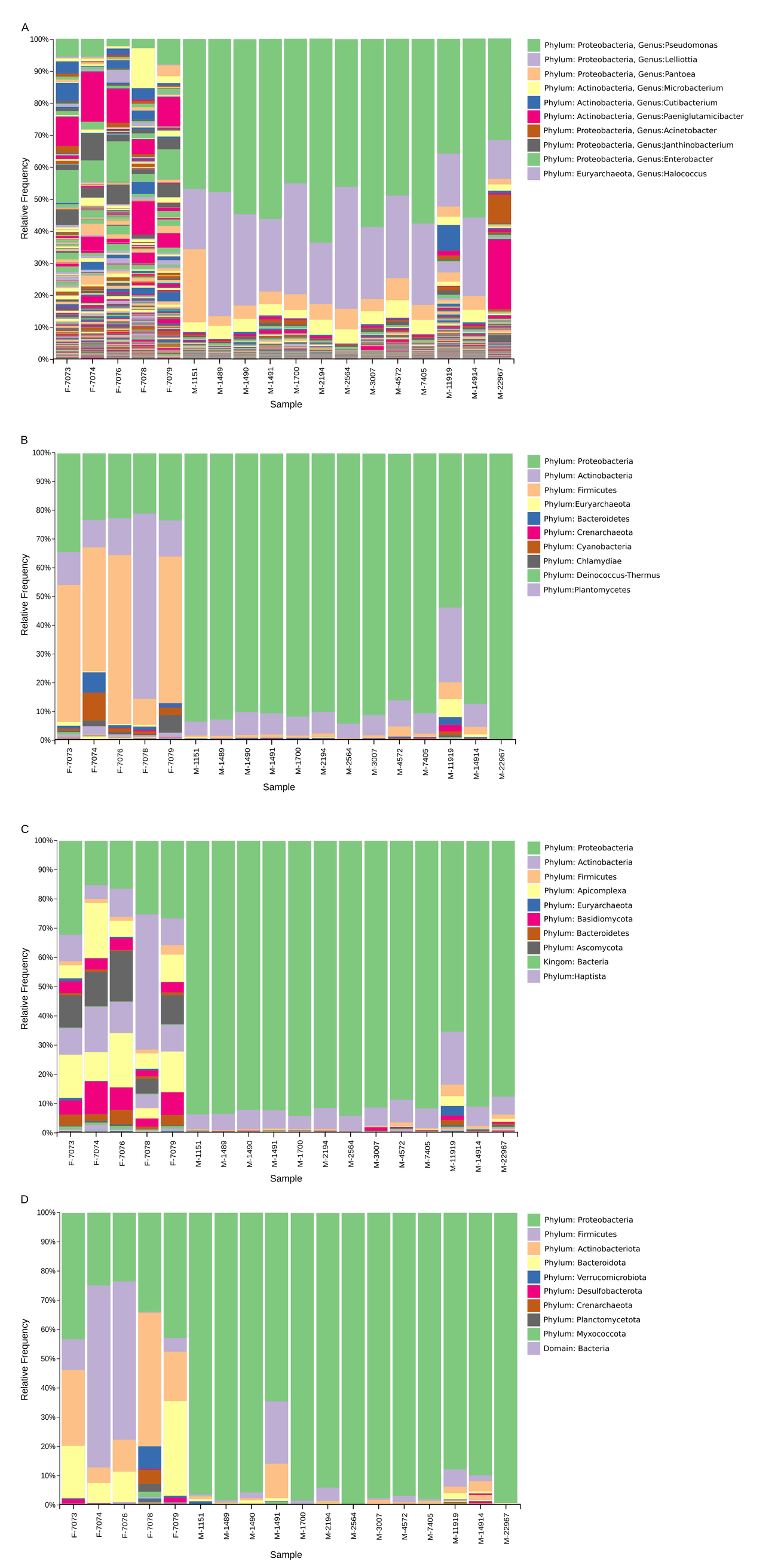

Supplement: S6 Fig — A–D. Abundance barplots. The top ten most commonly represented Phyla or Genera given in the legend. For taxa-level groupings not having a single known ID, the next highest known taxonomic level is given. Specimen ID is given for each column in addition to specimen type: F (fresh) or M (museum). (A) Abundance barplot of all specimen (n = 18) shotgun metagenomic sequencing derived taxonomies based on the Rep200 database. The top ten most commonly represented Genera are given in the legend. (B) Abundance barplot of all specimen (n = 18) shotgun metagenomic sequencing derived taxonomies based on the WoL database. The top ten most commonly represented Phyla are given in the legend. (C) Abundance barplot of all specimen (n = 18) shotgun metagenomic sequencing derived taxonomies based on the Rep200 database. The top ten most commonly represented Phyla are given in the legend. (D) Abundance barplot of all specimen (n = 18) 16S rRNA gene sequencing derived taxonomies based on the Greengenes database. The top ten most commonly represented Phyla are given in the legend. (TIF) [file pone.0291540.s006.tif]

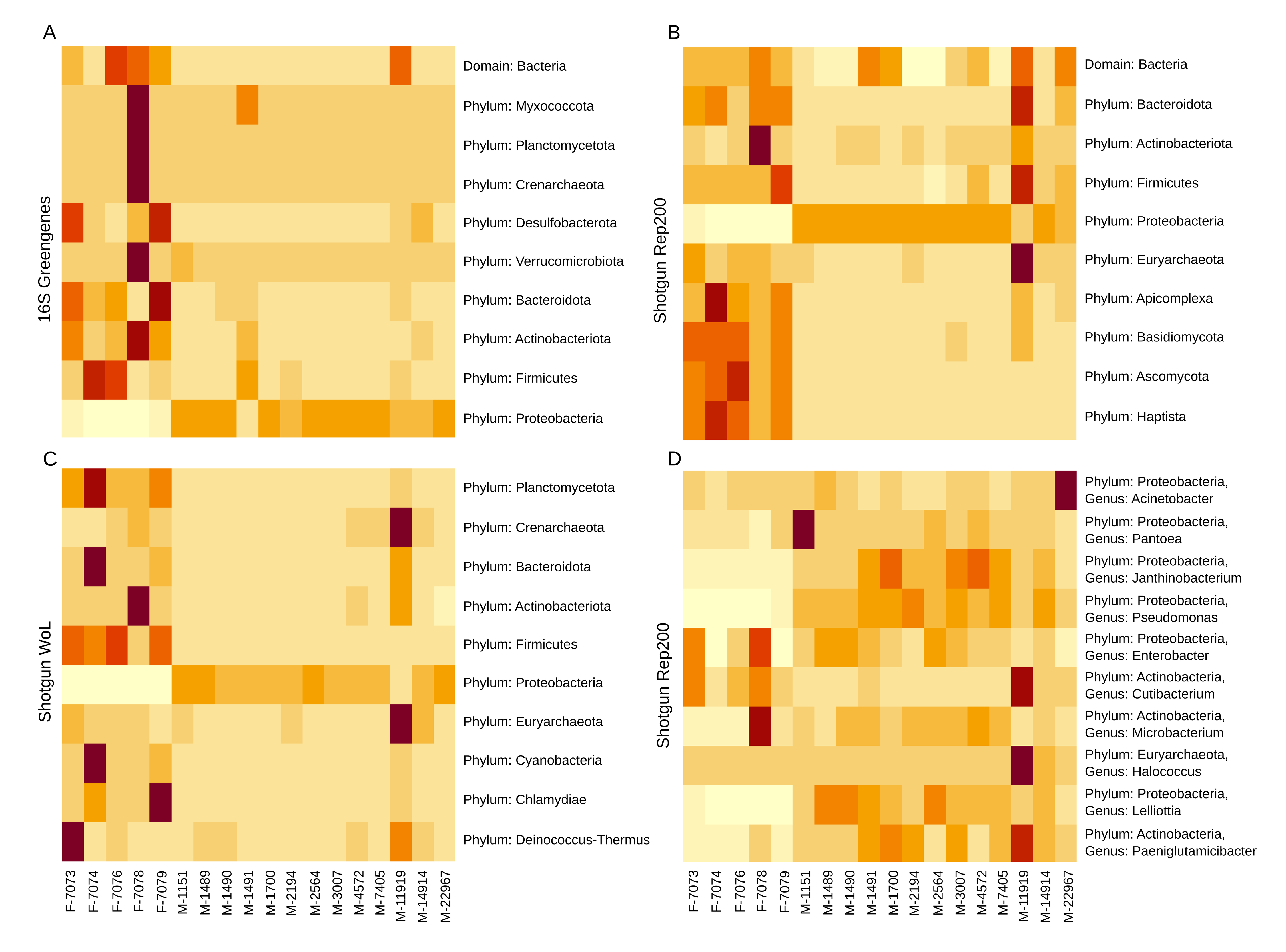

Supplement: S7 Fig — A–D. Heatmap of the ten (n = 10) most highly represented. (A) Phlya (of combined fresh and museum specimens) using 16S rRNA gene (Greengenes) sequencing (B) Phlya (of combined fresh and museum specimens) using Shotgun metagenomic (Rep200) sequencing (C) Phlya (of combined fresh and museum specimens) using Shotgun metagenomic (WoL sequencing (D) Genera (of combined fresh and museum specimens) using Shotgun metagenomic (Rep200) sequencing. Each row totals 100% (row normalized) with dark red approaching 100% and light yellow approaching 0%. For squares with <0.25%, 0 is assumed for purposes of visualization and normalization. For Phyla or Genus-level groupings not having a single known ID, the next highest known taxonomic level is given. Specimen ID is given for each column in addition to specimen type: F (fresh) or M (museum). (TIF) [file pone.0291540.s007.tif]
