# Supplementary material for: Shotgun metagenomics captures more microbial diversity than targeted 16S rRNA gene sequencing for field specimens and preserved museum specimens
Source: PLoS One. 2023 Sep 19;18(9):e0291540. doi: 10.1371/journal.pone.0291540 (PMC10508626; doi:10.1371/journal.pone.0291540)
Supplement: Attachment — Submitted filename: PONE-D-23-13742_review.pdf [file pone.0291540.s011.pdf]

PLOS ONE

Shotgun Metagenomics Describes Gut Microbiomes Better than Targeted 16S rRNA Gene Sequencing for Field Specimens and Preserved Museum Specimens

--Manuscript Draft--

**Manuscript Number:**	PONE-D-23-13742
**Article Type:**	Research Article
**Full Title:**	Shotgun Metagenomics Describes Gut Microbiomes Better than Targeted 16S rRNA Gene Sequencing for Field Specimens and Preserved Museum Specimens
**Short Title:**	Shotgun Metagenomics of Amphibian Museum Specimens
**Corresponding Author:**	Joseph Madison<br>UMass Boston: University of Massachusetts Boston<br>Boston, MA UNITED STATES
**Keywords:**	amphibian, gut microbiome, museum, preserved, shotgun, 16S
**Abstract:**	The use of museum specimens for research in microbial evolutionary ecology remains an under-utilized investigative dimension with important potential. Despite this potential, there remain barriers in methodology and analysis to the wide-spread adoption of museum specimens for such studies. Here, we hypothesized that there would be significant differences in taxonomic prediction and related diversity among sample type (museum or fresh) and sequencing strategy (medium-depth shotgun metagenomic or 16S rRNA gene). We found dramatically higher predicted diversity from shotgun metagenomics when compared to 16S rRNA gene sequencing in museum and fresh samples, with this differential being larger in museum specimens. Broadly confirming these hypotheses, the highest diversity found in fresh samples was with shotgun sequencing using the Rep200 reference inclusive of viruses and microeukaryotes, followed by the WoL reference database. In museum-specimens, community diversity metrics also differed significantly between sequencing strategies, with the alpha-diversity ACE differential being significantly greater than the same comparisons made for fresh specimens. Beta diversity results were more variable, with significance dependent on reference databases used. Taken together, these findings demonstrate important differences in diversity results and prompt important considerations for future experiments and downstream analyses aiming to incorporate microbiome datasets from museum specimens.
**Order of Authors:**	Joseph Madison
	Brandon LaBumbard
	Douglas Woodhams
**Additional Information:**	
**Question**	**Response**
**Financial Disclosure**<br><br>Enter a financial disclosure statement that describes the sources of funding for the work included in this submission. Review the submission guidelines for detailed requirements. View published research articles from *PLOS ONE* for specific examples.<br><br>This statement is required for submission and **will appear in the published article** if the submission is accepted. Please make sure it is accurate.	JDM is supported by the NSF Postdoctoral Research Fellowships in Biology Program (Award 1907311; www.nsf.gov) and DCW by NSF Career and Infrastructure Awards (Award IOS-845634 and BII 2120084; www.nsf.gov). Any opinions, findings, and conclusions or recommendations expressed in this material are those of the authors and do not necessarily reflect the views of NSF. NSF had no role in study design, data collection and analysis, decision to publish, or preparation of the manuscript.

Powered by Editorial Manager® and ProduXion Manager® from Aries Systems Corporation

**Unfunded studies**<br>Enter: *The author(s) received no specific funding for this work.*<br><br>**Funded studies**<br>Enter a statement with the following details:<br>• Initials of the authors who received each award<br>• Grant numbers awarded to each author<br>• The full name of each funder<br>• URL of each funder website<br>• Did the sponsors or funders play any role in the study design, data collection and analysis, decision to publish, or preparation of the manuscript?<br>• **NO** - Include this sentence at the end of your statement: *The funders had no role in study design, data collection and analysis, decision to publish, or preparation of the manuscript.*<br>• **YES** - Specify the role(s) played.<br><br>* typeset	
**Competing Interests**<br><br>Use the instructions below to enter a competing interest statement for this submission. On behalf of all authors, disclose any competing interests that could be perceived to bias this work—acknowledging all financial support and any other relevant financial or non-financial competing interests.<br><br>This statement is required for submission and **will appear in the published article** if the submission is accepted. Please make sure it is accurate and that any funding sources listed in your Funding Information later in the submission form are also declared in your Financial Disclosure statement.<br><br>View published research articles from *PLOS ONE* for specific examples.	The authors have declared that no competing interests exist.

**NO authors have competing interests**<br><br>Enter: *The authors have declared that no competing interests exist.*<br><br>**Authors with competing interests**<br><br>Enter competing interest details beginning with this statement:<br><br>*I have read the journal's policy and the authors of this manuscript have the following competing interests: [insert competing interests here]*<br><br>* typeset	
**Ethics Statement**<br><br>Enter an ethics statement for this submission. This statement is required if the study involved:<br><br>• Human participants<br>• Human specimens or tissue<br>• Vertebrate animals or cephalopods<br>• Vertebrate embryos or tissues<br>• Field research<br><br>Write "N/A" if the submission does not require an ethics statement.<br><br>General guidance is provided below. Consult the submission guidelines for detailed instructions. **Make sure that all information entered here is included in the Methods section of the manuscript.**	Specimens used in this study were sourced from the Milwaukee Public Museum Vertebrate Zoology Collection, the Illinois Natural History Survey Herpetology Collection, and the University of Illinois Museum of Natural History Amphibian and Reptile Collection. Fresh swabs used in this study were taken from voucher museum specimens collected by Drew R. Davis under a South Dakota Game, Fish and Parks Scientific Collector's Permit (#2020-2). Vouchering was completed using standard best practices under an approved IACUC protocol (AUP 18-28 issued to collaborator Drew R. Davis then at The University of Texas Rio Grande Valley, who performed the collecting as part of ongoing field surveys).

Powered by Editorial Manager® and ProduXion Manager® from Aries Systems Corporation

**Format for specific study types**<br><br>**Human Subject Research (involving human participants and/or tissue)**<br>• Give the name of the institutional review board or ethics committee that approved the study<br>• Include the approval number and/or a statement indicating approval of this research<br>• Indicate the form of consent obtained (written/oral) or the reason that consent was not obtained (e.g. the data were analyzed anonymously)<br><br>**Animal Research (involving vertebrate animals, embryos or tissues)**<br>• Provide the name of the Institutional Animal Care and Use Committee (IACUC) or other relevant ethics board that reviewed the study protocol, and indicate whether they approved this research or granted a formal waiver of ethical approval<br>• Include an approval number if one was obtained<br>• If the study involved *non-human primates*, add *additional details* about animal welfare and steps taken to ameliorate suffering<br>• If anesthesia, euthanasia, or any kind of animal sacrifice is part of the study, include briefly which substances and/or methods were applied<br><br>**Field Research**<br><br>Include the following details if this study involves the collection of plant, animal, or other materials from a natural setting:<br>• Field permit number<br>• Name of the institution or relevant body that granted permission	
**Data Availability**<br><br>Authors are required to make all data underlying the findings described fully available, without restriction, and from the time of publication. PLOS allows rare exceptions to address legal and ethical concerns. See the PLOS Data Policy and FAQ for detailed information.	Yes - all data are fully available without restriction

A Data Availability Statement describing where the data can be found is required at submission. Your answers to this question constitute the Data Availability Statement and **will be published in the article**, if accepted.<br><br>**Important:** Stating 'data available on request from the author' is not sufficient. If your data are only available upon request, select 'No' for the first question and explain your exceptional situation in the text box.<br><br>Do the authors confirm that all data underlying the findings described in their manuscript are fully available without restriction?	
**Describe where the data may be found in full sentences. If you are copying our sample text, replace any instances of XXX with the appropriate details.**<br><br>• If the data are **held or will be held in a public repository**, include URLs, accession numbers or DOIs. If this information will only be available after acceptance, indicate this by ticking the box below. For example: *All XXX files are available from the XXX database (accession number(s) XXX, XXX.).*<br>• If the data are all contained **within the manuscript and/or Supporting Information files**, enter the following: *All relevant data are within the manuscript and its Supporting Information files.*<br>• If neither of these applies but you are able to provide **details of access elsewhere**, with or without limitations, please do so. For example:<br><br>*Data cannot be shared publicly because of [XXX]. Data are available from the XXX Institutional Data Access / Ethics Committee (contact via XXX) for researchers who meet the criteria for access to confidential data.*<br><br>*The data underlying the results presented in the study are available from (include the name of the third party*	Relevant results data are within the manuscript and its Supporting Information files. The workflow used for processing and analysis within Qiita is available as a public study: 'Shotgun versus 16S - Museum and Fresh Leopard Frog Guts - ID 14549'. Additional code used for nucleic acid data processing and related statistical analyses are available in a GitHub repository at https://github.com/kvasir7/16S_shotgun_comparison_project. Raw sequencing files in fastq.gz format can be found on the Sequence Read Archive as BioProject PRJNA836960.

and contact information or URL).<br>• This text is appropriate if the data are owned by a third party and authors do not have permission to share the data.<br><br>* typeset	
Additional data availability information:	Tick here if the URLs/accession numbers/DOIs will be available only after acceptance of the manuscript for publication so that we can ensure their inclusion before publication.

Powered by Editorial Manager® and ProduXion Manager® from Aries Systems Corporation

Manuscript w/ Figures and Supplemental
Click here to access/download;Manuscript;Museum_Rana_pipiens_microbiom

Shotgun Metagenomics Describes Gut Microbiomes Better than Targeted 16S rRNA Gene Sequencing for Field Specimens and Preserved Museum Specimens

Joseph D. Madison[1†], Brandon C. LaBumbard[1], Douglas C. Woodhams[1]

[1]Department of Biology, University of Massachusetts Boston, Boston, MA, 02125, USA

[†]Correspondence can be addressed to JM at: josephdmadison7@gmail.com

Abstract

The use of museum specimens for research in microbial evolutionary ecology remains an under-utilized investigative dimension with important potential. Despite this potential, there remain barriers in methodology and analysis to the wide-spread adoption of museum specimens for such studies. Here, we hypothesized that there would be significant differences in taxonomic prediction and related diversity among sample type (museum or fresh) and sequencing strategy (medium-depth shotgun metagenomic or 16S rRNA gene). We found dramatically higher predicted diversity from shotgun metagenomics when compared to 16S rRNA gene sequencing in museum and fresh samples, with this differential being larger in museum specimens. Broadly confirming these hypotheses, the highest diversity found in fresh samples was with shotgun sequencing using the Rep200 reference inclusive of viruses and microeukaryotes, followed by the WoL reference database. In museum-specimens, community diversity metrics also differed significantly between sequencing strategies, with the alpha-diversity ACE differential being significantly greater than the same comparisons made for fresh specimens. Beta diversity results were more variable, with significance dependent on reference databases used. Taken together, these findings demonstrate important differences in diversity results and prompt important considerations for future experiments and downstream analyses aiming to incorporate microbiome datasets from museum specimens.

Keywords**: amphibian, gut microbiome, museum, preserved, shotgun, 16S**

Word Count (excluding tables and figures)**: 6052**

Introduction

The use of museum specimens for nucleic acid sequencing has great potential given the emergence of high-throughput molecular technologies (Card et al., 2021). These technologies applied to museum specimens can be used to ask questions of temporal relevance to ecology and evolution not otherwise accessible to experimental inquiry. Unfortunately, the conditions of specimen preservation techniques such as formalin fixation and storage in alcohol fluids (e.g. ethanol, isopropanol) have been a major hindrance to the use and full exploitation of these molecular technologies. This has resulted in a concerted effort to improve both wet-lab and downstream analytical methods for overcoming these challenges such that molecular technologies might be better used in conjunction with the vast catalogs of biological collections represented in museum repositories (Nakahama, 2021; Raxworthy & Smith, 2021; Derkarabetian, 2019).

One area of recent methodological focus for molecular analysis of preserved specimens is the specimen-associated microbial community or microbiome. Characterizing the microbiome of fluid-preserved museum specimens is a rapidly increasing area of focus due to its relevance in biology broadly, but especially in the disciplines of ecology and evolution. The most common way to characterize host-associated microbiomes is through nucleic acid isolation followed by 16S rRNA gene sequencing. However, there are limitations to 16S rRNA gene sequencing including irrelevance to host-associated taxonomic groups with differing marker genes (e.g. ITS in fungi; White, 1990), groups with no universal marker genes (e.g. viruses; Adriaenssens & Cowan, 2014) and bias in 16S rRNA gene copy number among different bacteria that is not easily corrected (Starke et al., 2021). Alternatively, the use of shotgun metagenomic sequencing and associated analytical tools has expanded with the advent of high-throughput sequencing that generates large numbers of sequence reads that might allow higher resolution analysis. Where long-read sequencing might otherwise be used, high-throughput short-read shotgun metagenomics

is useful in dealing with the highly degraded (i.e. short reads) and low-input characteristics of nucleic acids associated with museum specimens. Here, we compare both 16S rRNA gene sequencing and short-read shotgun metagenomics methods for examining museum specimen-associated gut microbiomes.

While there has been previous work demonstrating differences among these two sequencing technologies for microbial applications in fresh specimens (Durazzi et al., 2021; Brumfield et al., 2020; Tessler et al., 2017; Jovel et al., 2016), there has been no previous work that we are aware of examining taxonomic and diversity representation of these two sequencing methods for museum specimen derived microbiota. In this study, we therefore hypothesized that previous results from fresh specimens would extend to museum specimens, with differences in taxonomic predictions between sequencing technologies and related diversity analysis of gut-isolated microbial nucleic acids of fluid-preserved specimens of Northern leopard frogs, *Rana pipiens*. Additionally, we hypothesized that the differences in museum specimen derived results would diverge from the differences observed in freshly sampled specimens. Generally confirming these hypotheses, our corresponding results indicate differences in the sequencing approaches (16S rRNA gene vs shotgun metagenomics) and specimen types (museum vs. fresh) that should be considered in future study design and experimental contexts.

Materials and Methods

Specimen Origin and Storage

All museum specimens used in this study (n=13) were originally sourced from various locales in the Midwestern States of Wisconsin and Illinois, and obtained from the Milwaukee Public Museum or the Illinois Natural History Survey/University of Illinois Natural History Museum. The specimens used were originally field collected over a 120 year period with approximately one specimen per decade (1892–2012; see supplemental specimen metadata) to capture temporal degradation effects and likely differing storage

conditions common among many older museum specimens with uncertain storage histories. The fresh specimens (n=5) were from *R. pipiens* collected in eastern South Dakota, an area that encompasses the historic midwestern Tallgrass Prairie — a biome that extends through the aforementioned Wisconsin and Illinois regions (Helzer, 2009) and has historically similar soil microbial communities (Feirer, 2013). Fresh specimens were euthanized via topical application of 20% benzocaine. Specimens were collected under a South Dakota Game, Fish and Parks Scientific Collector's Permit (#2020-2) and IACUC protocol (AUP 18-28 issued to collaborator Drew R. Davis at The University of Texas Rio Grande Valley, who did the collecting).

All collection locales over both current and historical time periods captured in this study exhibit or have exhibited similar climatic patterns and land-use dominated by mono-crop agricultural operations. All museum specimens were received in ethanol and were stored on site at the University of Massachusetts Boston in the same ethanol concentrations as received, within individual sterilized mason jars (Ball). Jar size used was variable and dependent on specimen size. After initial receipt, specimens were kept in their respective jars until dissection.

Specimen Dissections and Swabbing

All dissections and swabbing were completed under aseptic conditions to include PPE and sterile equipment, where applicable. For the dissections, museum specimens were placed on 20.3 cm x 25.4 cm ABD sterile pads (McKesson Medical-Surgical Inc., Richmond, VA) within an aseptic Class II Type A2 Biological Safety Cabinet (Labgard ES, Energy Saver, Nuaire, Plymouth, MN). A 2 cm incision was then made in the ventral abdomen to expose the intestines (disposable gamma irradiated No. 12 scapels; Swann-Morton, Sheffield, England, UK). After pinning open the body cavity, a 1 cm longitudinal incision was made along the mid-intestine. The inside of the intestine was then swabbed (Rayon bud; Medical Wire Equipment, MW113, Corsham, Wiltshire, England, UK) by twisting the swab three times (1080 degrees). Specimens from fresh caught frogs were also obtained following the protocol above using

aseptic technique, with the exception that dissections were carried out in the field immediately following euthanasia, as opposed to dissection in a biosafety cabinet. The swab was next immediately placed in a micro-centrifuge tube for downstream processing (modified from Hykin et al., 2015) to include DNA extraction and sequencing.

Phenol-Chloroform DNA Extractions

Nucleic acid extractions were completed using a modified phenol-chloroform extraction for museum samples as per Campos & Gilbert (2012). Briefly, swabs were incubated in 500 µL of alkaline digestion buffer for 40 min at 100°C on a heating block (Drybath Standard 2-block, Thermo Scientific). The alkaline buffer was composed of Sodium dodecyl sulfate (BioXtra ≥ 99.0%, Sigma-Aldrich, St. Louis, MO) and Sodiumhydroxide solution from a 10 M stock aq. (BioUltra, Sigma-Aldrich). After cooling to room-temperature, 500 µL of phenol-chloroform-isoamyl alcohol mixture solution was added (25:24:1; BioUltra, Sigma-Aldrich). The solution was then agitated on a shaker table (Lab-line Maxirotator, Lab-Line Instruments Incorporated, Melrose Park, IL) for 5 min on the high setting. The solution was then centrifuged for 5 min at 12,000 x g (~rcf) (Centrifuge 5425, Eppendorf, Hamburg, Germany). After centrifugation, the top aqueous layer was added to 500 µL of chloroform (≥ 99.5% containing 100–200 ppm amylenes as stabilizer; Sigma-Aldrich) in a new 1.5 mL microcentrifuge tube. This was followed by another round of centrifugation for 5 min at 12,000 x g (~rcf). The top layer was then added to a new 1.5 mL centrifuge tube, followed by addition of 300 µL (0.7 volume) 2-propanol (BioReagent for molecular biology ≥99.5%, Sigma-Aldrich) and 50 µL (~0.1 volume) of molecular biology grade 3 M sodium acetate at pH 5.2 (EMD Millipore Corp., Billerica, MA). This mixture was then centrifuged for 30 min at 12,000 x g (~rcf). Following the 30 min centrifugation, the liquid was decanted and disposed. The pellet was then washed by adding 500 µL of ethanol (85% aq.; 200 proof molecular biology grade ethyl alcohol, Sigma-Aldrich, diluted with UltraPure distilled water, (Invitrogen, Grand Island, NY), inverting once, and centrifugation for 5 min at 12,000 x g (rcf). After this centrifugation, the ethanol solution was decanted

and disposed, with any remaining solution removed with a small diameter pipette. The tubes were then briefly incubated at 70°C to remove any residual ethanol. Lastly, the pellet was re-suspended by adding 50 μL of 1X Tris-EDTA buffer solution at pH 8.0 (BioUltra, Sigma-Aldrich) and mixing with a pipette. The suspended nucleic acids were then stored in a 4°C refrigerator or -80°C freezer depending on the immediacy of downstream applications.

Shotgun Metagenomics Sequencing and Analysis

Library preparation for sequencing reactions was carried out using an NEB workflow for the NEBNext Ultra II DNA library prep kit for Illumina (New England Biolabs, Ipswich, MA). Paired-end 151 bp sequencing was carried out by the University of Minnesota Genomics Core on an Illumina NovaSeq 6000 with an average read depth of 23,844,08 reads/sample (range: 9108413–54875278/sample; see Supplemental Table 1).

For analysis, raw Illumina output was first sorted by index-linked sample ID. Sorted reads were then quality inspected with FastQC followed by host and background nucleic acid removal. This was completed by construction of custom host databases of *R. temporaria* and *B. Bufo* with Kraken2 (Wood et al., 2019). Non-matching reads to the host database meeting the 0.5 confidence threshold were then sorted into a separate file for downstream analysis. The *R. temporaria* genome was used as a completed whole-genome reference is not available for *R. pipiens* (the host from which swabs were derived). The *R. temporaria* genome has high homology to *R. pipiens,* and the same karyotype (Perkins et al., 2019), making it an acceptable reference for this filtering step. Likewise, sequences with 0.5 confidence homology to *Bufo bufo* were matched and removed as separate *Anaxyrus americanus* museum specimens were present and processed in the laboratory space used.

Background swabs processed in parallel but without specimen swabbing were also used as controls for background contamination removal. Following the same procedure as the host removal, a database was constructed in Kraken2 and matches were removed. A more conservative confidence

threshold of 0.8 was used so as to ameliorate concerns of erroneous removal of close matches between bacteria in the sample and background (Supplementary Figure 1). Exact matches resulting in sample removal were unlikely based on differential degradation patterns in the specimen and also visual inspection of sequence taxonomy tables. After quality inspection, host removal, and background removal, the output was analyzed in the Qiita metagenomic analysis pipeline (Gonzalez et al., 2018) and is available in the Data Accessibility and Benefit-Sharing section below.

Qiita analysis followed the recommended shotgun metagenomics pipeline. This workflow includes an initial sample adapter removal step with fastp (Chen et al., 2018), human host filtering with minimap2 (to account for sample contamination during handling; Li, 2018), and taxonomic profiling via bowtie2 (Langmead & Salzberg, 2012) with either the WoL reference database (Zhu et al., 2019; representing Bacteria and Archaea) or the Rep 200 database which is composed of RefSeq assemblies (Al-Ghalith & Knights, 2020; representing Archaea, Bacteria, Fungi, Protozoa, and Viruses), and feature tables generated with Woltka (Zhu et al., 2022). Woltka output was analyzed with QIIME2 (Bolyen et al. 2018). Shotgun metagenomic data was not subject to rarefaction prior to downstream diversity analyses due to qualitatively increasing alpha diversity with increasing sequence depth (no plateau), and also greater uncertainty as to the effects of rarefaction on analysis of shotgun metagenomic sequencing data (as opposed to 16S rRNA gene sequencing).

16S rRNA Gene Sequencing and Analysis

After DNA extractions (using the same protocol and from the same tubes containing nucleic acids used in shotgun metagenomic sequencing), a subset of nucleic acids was also used for 16S rRNA gene sequencing. PCR was conducted in duplicate to amplify the V4 region of the 16S rRNA bacterial gene (515F and 806R primers) following the Earth Microbiome protocol (Caporaso et al. 2012). Following PCR, sample amplicons were pooled and then purified and normalized using a Mag-Bind EquiPure Library Normalization Kit (Omega Bio-tek, Inc., Norcross, GA, USA). The library consisted of 10 μL of

each normalized sample pooled together, which was then sequenced on an Illumina MiSeq v2 300 cycles cartridge.

The raw Illumina 16S rRNA amplicon data (Supplemental Table 2) was processed and quality filtered using QIIME 2 v2020.2 (Bolyen et al. 2018) and classified into amplicon sequence variants (ASVs) using the DADA2 workflow (Callahan et al. 2016). Within DADA2, reads were trimmed to 150 bp based on quality score checks for all samples (Supplementary Figure 2., A–R), and bacterial taxonomy was assigned using 16S Greengenes 13_8 99% OTUs (McDonald et al. 2012) reference classifier (Bacteria and Archaea). ASV reads found in extraction and PCR negative controls with more than 20 reads were deemed as contaminants and filtered out of all samples. We also filtered out reads assigned as "mitochondria" and "chloroplast". Next the dataset was rarefied at 3000 to normalize read counts across samples. We subsequently generated several metrics on the dataset to analyze differences in alpha (ACE and Shannon's) and beta (Jaccard) diversity. These microbiome diversity metrics and related taxonomic summaries were determined using QIIME2 for downstream statistical analyses. For visualization, we used both the EMPeror visualization tool and innate R plotting for illustrating trends, and to describe statistical differences among methods as given below.

Statistical Analyses

All statistical analyses to include alpha and beta diversity comparisons were performed either within Qiita/QIIME2 or in R (R Core Team 2020). Alpha diversity was examined between shotgun metagenomic and targeted 16S rRNA gene sequencing methods, and between museum-preserved and fresh samples. Two alpha diversity indices were used in these comparisons: Shannon's diversity index (Shannon & Weaver, 1949) and the abundance-based coverage estimator (ACE) metric. Shannon diversity was used as it is a commonly used diversity metric and thus broadly interpretable. ACE was developed as an improvement on the Chao1 index (Chao & Yang, 1993) and is used due to its ability to correct for low

represented community members and thus underrepresented abundances; a phenomena we expected from using degraded nucleic acids from preserved museum specimens.

Beta diversity and related matrix comparisons were also performed between both sequence methods (shotgun metagenomic or 16S rRNA gene) and sampling methods (museum or fresh collected). Full mantel tests and procrustes analysis on the derived Jaccard distance matrices and related principle coordinate analyses (PCOA) were used to test for matrix distance similarity and microbial community structure, respectively. Mantel tests were performed to test the null hypothesis that there is a lack of relationship between values in pairwise dissimilarity matrix comparisons. This technical question arises from the methodological use of distance matrices and their derivation method (in this case sequencing strategies), which is different from the rationale criticized by Legendre et al. (2015) in landscape ecology studies.

We also qualitatively examined taxonomic differences between sequencing methods and sample types (museum or fresh). Taxonomy plots of fresh and specimen derived samples were made for 16S rRNA gene sequencing derived ASVs and of shotgun metagenomic sequencing derived species-level taxa generated from alignment-based matching with Rep 200 or WoL. Heatmaps were also made of the top-ten most represented phyla and genera among the three sequencing comparisons made (with fresh and museum combined, grouped by either phyla or genera as seen in the corresponding figures).

Results

Community alpha and beta diversity metrics were examined for comparing shotgun metagenomic (WoL and Rep200 reference databases) and 16S rRNA gene sequencing methods (Greengenes). These metrics indicate significant differences in the predictions of shotgun metagenomic and 16S rRNA gene sequencing

methods, with more limited differences observed between the different shotgun metagenomic sequencing reference databases used.

Alpha Diversity Analysis

The omnibus Kruskal-Wallis tests for both alpha diversity metrics (Shannon's diversity index and ACE) indicate significant differences among shotgun metagenomic sequencing data classified with WoL, shotgun metagenomic sequencing classified with rep200, and 16S rRNA gene sequencing data classified with Greengenes ($p<0.05$; $\alpha=0.05$; Tables 1A and 2A). Posthoc Dunn's (nonparametric) tests for both diversity metrics (Shannon's diversity index and ACE) indicate varied results depending on the reference database used (Tables 1B and 2B). Of note, the museum specimens were significantly different among all pairwise comparisons examined for both Shannon's diversity index and ACE (Benjamini-Hochberg-adjusted $p<0.05$; $\alpha=0.05$). Pairwise comparisons between fresh specimen methods varied. The shotgun metagenomic (Rep200)-16S rRNA gene (fresh) comparison was significantly different for both diversity metrics whereas the shotgun metagenomic (WoL)-16S rRNA gene (fresh) comparison was not significantly different ($p>0.05$; $\alpha=0.05$) for both diversity metrics. The between shotgun metagenomic comparisons of WoL and Rep200 (fresh) were not significantly different for ACE ($p=0.232$, $\alpha=0.05$), but were significantly different for Shannon's diversity ($p=0.0216$, $\alpha=0.05$). The respective alpha diversity boxplots qualitatively compare the shotgun metagenomic sequencing data classified with the Rep200 database, shotgun metagenomic sequencing data classified with the WoL database, and 16S rRNA gene sequencing results classified with Greengenes (Figures 1A–D).

Mantel Tests and Procrustes Analysis

Mantel tests and Procrustes analysis of fresh and museum-preserved specimens indicate insignificant statistical correlations (i.e. there are differences between methods) between 16S rRNA gene (Greengenes) and shotgun metagenomic sequencing (both Rep200 and WoL) methods. Specifically, Mantel test Spearman's correlations (ρ) ranged from 0.139–0.358 (permutations = 999 for all comparisons) and

associated p-values for all comparisons ranged from 0.158–0.711 (Figure 2 A–D). These results indicate no significant differences in Jaccard matrix distance correlations between 16S rRNA gene and shotgun metagenomic sequencing among the tested pairwise comparisons.

Additionally, Procrustes analysis (Figure 3 A–D) indicate significant differences between 16S and shotgun metagenomic results for museum but not fresh specimens. For fresh specimens both the 16S rRNA gene-WoL and 16S rRNA gene-Rep200 comparisons were insignificant ($p>0.05$; $\alpha=0.05$). For museum specimens both the 16S rRNA gene-WoL and 16S rRNA gene-Rep200 comparisons were significant ($p=0.019$ (of true M^2) , $p= 0.007$ (of true M^2), respectively; $\alpha=0.05$). However, the 16S rRNA gene-WoL and 16S rRNA gene-Rep200 comparisons also had high M^2 values that could be interpreted as diminishing the significance of these results (True $M^2 = 0.520$, True $M^2 = 0.481$, respectively), as low M^2 values are generally needed to corroborate significant p-values. For the purposes of this study, they are interpreted as significant in the context of other statistical results presented.

Taxonomic Profiling

Taxa abundance bar plots indicate qualitative differences in taxa prediction between sequencing method (16S/Greengenes, shotgun/WoL, and shotgun/rep200) and specimen type (museum and fresh specimens of *R. pipiens)*. These qualitative differences indicate a general trend towards predictive divergence with increasing taxonomic resolution.

When examining Phyla-level resolution, all sequencing methods (16S/Greengenes, shotgun/WoL, and shotgun/rep200) have substantial overlap in their taxonomic predictions, with the Phyla Proteobacteria (Pseudomonodata), Actinobacteria, and Firmicutes being overrepresented as comp16ared to other Phyla (Supplementary Figures 3, 4, and 5). This trend is generally exhibited for both fresh specimens and museum specimens.

Changing the taxonomic classification to Genus-level resolution indicates wider discrepancies between sequencing methods and the fresh versus museum specimen comparison. Specifically, 16S rRNA

gene sequencing showed three Genera dominating the total community abundance in the museum specimens, whereas fresh specimens had more unique moderately represented Genera (Figure 4). This trend also held with shotgun metagenomic sequencing when comparing museum and fresh specimens with the Rep200 database (Figure 6). The WoL database also showed few dominating Genera for both the fresh and museum specimens (Figure 5), although these were classified differently, with Acinetobacter highly represented among the fresh specimens and Pseudomonas and Enterobacter highly represented among the museum specimen communities. Pseudomonas, Enterobacter, Acinetobacter, and Pantoea were however predicted as top ten Genera among all of the sequencing/analysis methods (Figures 4—7). Additionally, both shotgun metagenomic (WoL) and 16S rRNA gene sequencing methods exhibit concurrent sensitivity to a high prevalence of Rickettsiella in specimen 22967, which is an outlier from the other museum specimens.

In addition to bacterial predictions, all databases used had Archaea present which was well represented among samples examined by both 16S rRNA gene and shotgun metagenomic sequencing, and also among both fresh and museum specimens. The presence of Euryarchaeota was confirmed from both the WoL and rep200 comparisons of the shotgun metagenomic sequencing data, which is in agreement with previously described amphibian samples (Youngblut et al. 2021) and possibly ubiquitous among amphibians. Other Phylum-level Archaeal identification included Crenarchaeota, of which there was agreement on presence in the top ten most abundant Phyla between 16S rRNA gene (Greengenes) and shotgun metagenomic (WoL), with presence also confirmed in one sample by shotgun sequencing with Rep200 (although not in the top ten most represented). Genus-level taxonomic identification varies more considerably among sequencing methods and databases. Results other than Bacteria and Archaea were also produced by the shotgun metagenomics data categorized with Rep200 database. These results included limited classification of viruses, Protists, and Fungi (see supplemental taxonomy files).

Discussion

In this study, we examined differences in 16S rRNA gene and shotgun metagenomic sequencing derived nucleic acid datasets from freshly sampled and preserved museum specimens, hypothesizing that there would be differences between sequencing methods and between specimen type (museum or fresh). We confirmed our hypotheses by finding differences in diversity metrics and taxonomic predictions between 16S rRNA gene and shotgun metagenomic sequencing methods in both fresh and museum specimens, but with more pronounced differences in museum specimens. Alpha and beta diversity metric predictions and related statistical tests also exhibited some overlap between 16S rRNA gene and shotgun metagenomic sequencing methods in museum and fresh specimens among highly represented taxa.

Alpha diversity metrics differed significantly depending on the reference methods used, with museum specimens exhibiting significant differences between 16S rRNA gene and shotgun metagenomic sequencing (WoL and Rep200) for both diversity metrics (Shannon and ACE). Significant differences among fresh specimens were neither uniform nor as pronounced as museum specimens, and depended on the comparison being made and diversity metric chosen. The lack of significant alpha diversity differences seen in the shotgun metagenomic (WoL)-16S rRNA gene (fresh) comparison indicate that comparisons of gut microbiome datasets using differing sequencing methods are at times appropriate. This finding does not, however, hold for the museum-derived microbiome samples. Mantel tests and Procrustes analysis of Jaccard distance matrices and derived PCoA results also indicate technical significance in the data generated by the compared methods. Specifically, Mantel tests showed no significant correlations among compared distances matrices, indicating differences between 16S rRNA gene and shotgun metagenomic methods for both fresh and museum-derived samples. Procrustes analysis also indicates significant differences among method for the museum specimens, but not for the fresh specimens.

In this study, the comparison of 16S rRNA gene and shotgun metagenomic derived data presented challenges for equitable comparison. A major consideration was in deciding which diversity metrics and reference databases to use for taxonomic comparison of the different datasets, specifically in referenced sequence abundance. To account for differences in abundance calculations of shotgun metagenomic and 16S rRNA gene sequencing, the Jaccard index of dissimilarity was used for efficacy of comparisons between sequencing strategies. This allowed taxonomic presence/absence determination between both strategies, as compared to abundance-linked diversity determinations (e.g. evenness in Shannon index). Abundance based diversity comparisons require correction or normalization procedures that are highly non-trivial when sourcing data or reference standards, and were outside of the scope of this study yet of interest for future work. The difficulty for correction or normalization arises due to copy numbers of sequences from 16S V4 sequences and those of taxonomically linked genomic regions represented in shotgun metagenomic sequencing data having uncorrelated or stochastic relationships. Where these abundance differences occur (and thus downstream diversity predictions), both methods would still be represented as either "present" or "absent" for the referenced taxa using the Jaccard index of dissimilarity, regardless of abundance. The one caveat to this are those taxa that are only present in one method and not the other, which represents a quantifiable difference in the scheme utilized and presented in this study as results. For the purposes of this study, we therefore constrained our beta diversity comparisons to the Jaccard's index of dissimilarity as a diversity measure.

Similar considerations for taxa under-representation due to the degraded nature of museum-sourced nucleic acid datasets were made for the chosen alpha diversity metrics. Specifically, the ACE index was used which is designed to account for low abundance or under-representation applications. This was in addition to the commonly used Shannon's diversity index, which showed differences from ACE in a subset of comparisons and was thus indicative of the importance of alpha diversity metric choice when using degraded nucleic acids (such as those from fluid-preserved museum specimens).

The results of this study represent the first comparison of museum-derived microbiome data with two commonly used sequencing strategies for such analysis: targeted 16S rRNA gene sequencing and medium-depth shotgun metagenomic sequencing. Multiple reference database and analysis procedures were also compared and represent those commonly used in microbiome studies. As this was only a preliminary analysis of differences between sequencing types in museum specimen microbiomes, future work should aim to include samples from different Chordate groups (e.g. fish) which may vary in their preservation methods and subsequently have different results than those for amphibians. As sequencing technologies change and allow greater depth and lower costs, comparisons such as this should continue to ensure inter-method comparisons are justified and that the best methods are used. For those research questions that are focused on microbial community ecology and evolution spanning host-associated microorganisms outside of Bacteria and Archaea (i.e. 16S), shotgun metagenomic sequencing might be the preferred method due to the broad nucleic acid capture, and recognition of taxa other than Bacteria and Archaea (as seen with the Rep200 database used in this study generating limited results for viruses, fungi, and protozoa). Differences will also be dependent on the database used for taxonomic reconstruction or any downstream functional inference, such as the difference in represented taxa in the Rep200 (Archaea, Bacteria, Fungi, Protozoa, and viruses), WoL (Bacteria and Archaea), and Greengenes (Bacteria and Archaea) databases compared in this study. However, it is also important to make clear that the different Domains of taxa represented does not necessarily mean significant differences in downstream diversity comparisons, as was the case for the Rep200 – WoL fresh specimen comparison for ACE diversity.

Taken together, these results indicate that sequencing method choice is an important consideration when examining museum specimens—even more so than fresh specimens. These differences are in general agreement with previous work examining 16S rRNA gene and shotgun metagenomic sequencing methods showing differences between these methods and the commonly used downstream analysis

pipelines (Toole et al., 2021; Brumfield et al., 2020; Jovel et al., 2016). The work presented here is therefore important in including museum specimens as a sample type that follows this broader trend.

Differences in results between these two sequencing methods will also be important in future efforts to systematically integrate molecular datasets with associated specimens in museum repositories (Mulcahy et al., 2022). How interpretation of any such integration will occur should be thought about carefully due to implications including policy determination (Jensen et al., 2022) and the trajectory of future studies that will build upon results of initial museum-associated microbiome studies.

Acknowledgements

JDM is supported by the NSF Postdoctoral Research Fellowships in Biology Program (Award 1907311) and DCW by NSF Career and Infrastructure Awards (Award IOS-845634 and BII 2120084). Any opinions, findings, and conclusions or recommendations expressed in this material are those of the authors and do not necessarily reflect the views of NSF. Specimens used in this study were sourced from the Milwaukee Public Museum Vertebrate Zoology Collection, the Illinois Natural History Survey Herpetology Collection, and the University of Illinois Museum of Natural History Amphibian and Reptile Collection. Fresh samples were received from collection and subsequent dissection completed by Drew R. Davis. Additionally, JM is thankful for advice and insights received that improved this manuscript during his time as a visiting scholar in the Knight Lab at the University of California, San Diego.

References

Adriaenssens, E. M., & Cowan, D. A. (2014). Using signature genes as tools to assess environmental viral ecology and diversity. *Applied and environmental microbiology*, *80*(15), 4470—4480.

Amir, A., McDonald, D., Navas-Molina, J. A., Kopylova, E., Morton, J. T., Zech Xu, Z., ... & Knight, R. (2017). Deblur rapidly resolves single-nucleotide community sequence patterns. *MSystems*, *2*(2), e00191—16.

Al-Ghalith, G., & Knights, D. (2020). BURST enables mathematically optimal short-read alignment for big data. *BioRxiv*.

Bolyen, E., J. R. Rideout, M. R. Dillon, N. A. Bokulich, C. Abnet, G. A. Al-Ghalith, H. Alexander, E. J. Alm, M. Arumugam, and F. Asnicar. 2018. QIIME 2: Reproducible, interactive, scalable, and extensible microbiome data science. 2167—9843, PeerJ Preprints.
Brumfield, K. D., Huq, A., Colwell, R. R., Olds, J. L., & Leddy, M. B. (2020). Microbial resolution of whole genome shotgun and 16S amplicon metagenomic sequencing using publicly available NEON data. *PLoS One*, *15*(2), e0228899.
Callahan, B. J., McMurdie, P. J., Rosen, M. J., Han, A. W., Johnson, A. J. A., & Holmes, S. P. (2016). DADA2: High-resolution sample inference from Illumina amplicon data. *Nature methods*, *13*(7), 581—583.
Campos, P. F., & Gilbert, T. M. (2012). DNA extraction from formalin-fixed material. In Ancient DNA (pp. 81—85). Humana Press.
Caporaso, J. G., C. L. Lauber, W. A. Walters, D. Berg-Lyons, J. Huntley, N. Fierer, S. M. Owens, J. Betley, L. Fraser, and M. Bauer. 2012. Ultra-high-throughput microbial community analysis on the Illumina HiSeq and MiSeq platforms. The ISME journal **6**:1621—1624.
Card, D. C., Shapiro, B., Giribet, G., Moritz, C., & Edwards, S. V. (2021). Museum genomics. *Annual review of genetics*, *55*, 633—659.
Chao, A., & Yang, M. C. (1993). Stopping rules and estimation for recapture debugging with unequal failure rates. *Biometrika*, *80*(1), 193—201.
Chen, S., Zhou, Y., Chen, Y., & Gu, J. (2018). fastp: an ultra-fast all-in-one FASTQ preprocessor. *Bioinformatics*, *34*(17), i884—i890.
Derkarabetian, S., Benavides, L. R., & Giribet, G. (2019). Sequence capture phylogenomics of historical ethanol- preserved museum specimens: Unlocking the rest of the vault. *Molecular ecology resources*, *19*(6), 1531—1544.
Durazzi, F., Sala, C., Castellani, G., Manfreda, G., Remondini, D., & De Cesare, A. (2021). Comparison between 16S rRNA and shotgun sequencing data for the taxonomic characterization of the gut microbiota. *Scientific reports*, *11*(1), 1—10.
Fierer, N., Ladau, J., Clemente, J. C., Leff, J. W., Owens, S. M., Pollard, K. S., ... & McCulley, R. L. (2013). Reconstructing the microbial diversity and function of pre-agricultural tallgrass prairie soils in the United States. *Science*, *342*(6158), 621—624.
Gonzalez, A., Navas-Molina, J. A., Kosciolek, T., McDonald, D., Vázquez-Baeza, Y., Ackermann, G., ... & Knight, R. (2018). Qiita: rapid, web-enabled microbiome meta-analysis. *Nature methods*, *15*(10), 796—798.
Helzer, C. (2009). *The ecology and management of prairies in the central United States*. University of Iowa Press.
Hykin, S. M., Bi, K., & McGuire, J. A. (2015). Fixing formalin: a method to recover genomic-scale DNA sequence data from formalin-fixed museum specimens using high-throughput sequencing. *PloS one*, *10*(10), e0141579.
Jensen, E. L., Díez-del-Molino, D., Gilbert, M. T. P., Bertola, L. D., Borges, F., Cubric-Curik, V., ... & Vernesi, C. (2022). Ancient and historical DNA in conservation policy. *Trends in Ecology & Evolution*.
Jovel, J., Patterson, J., Wang, W., Hotte, N., O'Keefe, S., Mitchel, T., ... & Wong, G. K. S. (2016). Characterization of the gut microbiome using 16S or shotgun metagenomics. *Frontiers in microbiology*, *7*, 459.
Langmead, B., & Salzberg, S. L. (2012). Fast gapped-read alignment with Bowtie 2. *Nature methods*, *9*(4), 357—359.
Li, H. (2018). Minimap2: pairwise alignment for nucleotide sequences. *Bioinformatics*, *34*(18), 3094—3100.

Mulcahy, D. G., Ibáñez, R., Jaramillo, C. A., Crawford, A. J., Ray, J. M., Gotte, S. W., ... & de Queiroz, K. (2022). DNA barcoding of the National Museum of Natural History reptile tissue holdings raises concerns about the use of natural history collections and the responsibilities of scientists in the molecular age. *PloS one*, *17*(3), e0264930.

Legendre, P., Fortin, M. J., & Borcard, D. (2015). Should the Mantel test be used in spatial analysis?. *Methods in Ecology and Evolution*, *6*(11), 1239—1247.

Nakahama, N. (2021). Museum specimens: An overlooked and valuable material for conservation genetics. *Ecological Research*, *36*(1), 13—23.

Perkins, R. D., Gamboa, J. R., Jonika, M. M., Lo, J., Shum, A., Adams, R. H., & Blackmon, H. (2019). A database of amphibian karyotypes. *Chromosome Research*, *27*(4), 313—319.

Quast, C., E. Pruesse, P. Yilmaz, J. Gerken, T. Schweer, P. Yarza, J. Peplies, and F. O. Glockner. 2013. The SILVA ribosomal RNA gene database project: improved data processing and web-based tools. Nucleic Acids Res **41**:D590—596.

R Core Team. 2020. R: A language and environment for statistical computing. R Foundation for Statistical Computing, Vienna, Ausria.

Raxworthy, C. J., & Smith, B. T. (2021). Mining museums for historical DNA: advances and challenges in museomics. *Trends in Ecology & Evolution*, *36*(11), 1049-1060.

Shannon, C. E., & Weaver, W. (1949). The mathematical theory of communication. University of Illinois. *Urbana*, *117*.

Starke, R., Pylro, V. S., & Morais, D. K. (2021). 16S rRNA gene copy number normalization does not provide more reliable conclusions in metataxonomic surveys. *Microbial ecology*, *81*(2), 535—539.

Tessler, M., Neumann, J. S., Afshinnekoo, E., Pineda, M., Hersch, R., Velho, L. F. M., ... & Brugler, M. R. (2017). Large-scale differences in microbial biodiversity discovery between 16S amplicon and shotgun sequencing. *Scientific reports*, *7*(1), 1—14.

Toole, D. R., Zhao, J., Martens-Habbena, W., & Strauss, S. L. (2021). Bacterial functional prediction tools detect but underestimate metabolic diversity compared to shotgun metagenomics in southwest Florida soils. *Applied Soil Ecology*, *168*, 104129.

White, T. J., Bruns, T., Lee, S. J. W. T., & Taylor, J. (1990). Amplification and direct sequencing of fungal ribosomal RNA genes for phylogenetics. *PCR protocols: a guide to methods and applications*, *18*(1), 315—322.

Wood, D. E., Lu, J., & Langmead, B. (2019). Improved metagenomic analysis with Kraken 2. *Genome biology*, *20*(1), 1—13.

Youngblut, N. D., Reischer, G. H., Dauser, S., Maisch, S., Walzer, C., Stalder, G., ... & Ley, R. E. (2021). Vertebrate host phylogeny influences gut archaeal diversity. *Nature microbiology*, *6*(11), 1443—1454.

Zhu, Q., Mai, U., Pfeiffer, W., Janssen, S., Asnicar, F., Sanders, J. G., ... & Knight, R. (2019). Phylogenomics of 10,575 genomes reveals evolutionary proximity between domains Bacteria and Archaea. *Nature communications*, *10*(1), 1—14.

Zhu Q, Huang S, Gonzalez A, McGrath I, McDonald M, Haiminen N, Armstrong G, et al. Phylogeny-aware analysis of metagenome community ecology based on matched reference genomes while bypassing taxonomy. *mSystems*. 2022. e00167—22.

Data Accessibility

The workflow used for processing and analysis within Qiita is available as a public study: 'Shotgun versus 16S - Museum and Fresh Leopard Frog Guts - ID 14549'. Additional code used for nucleic acid data processing and related statistical analyses are available in a GitHub repository at https://github.com/kvasir7/16S_shotgun_comparison_project. Raw sequencing files in fastq.gz format can be found on the Sequence Read Archive as BioProject PRJNA836960.

Author Contributions

JM designed research, performed research, analyzed data, and wrote the paper. BL designed research, performed research, analyzed data, and wrote the paper. DW designed research, contributed reagents and tools, analyzed data, and wrote the paper.

Conflicts of Interest

The authors have no conflicts of interest to declare.

Ethics Statement

Fresh swabs used in this study were taken from voucher museum specimens collected by DRD under a South Dakota Game, Fish and Parks Scientific Collector's Permit (#2020-2). Vouchering was completed using standard best practices under an approved IACUC protocol (AUP 18-28 issued to collaborator Drew R. Davis at The University of Texas Rio Grande Valley, who did the collecting).

Tables

Table 1A–B. ACE comparisons between the six reference processing types using Kruskal-Wallis rank sum test followed by Dunn's post-hoc pairwise testing (Benjamini-Hochberg corrected).

A. Kruskal-Wallis Rank Sum Test

Comparison	Kruskal-Wallis chi-squared	df	P-value
Museum specimens	30.917	2	1.93e-07
Fresh specimens	11.324	2	0.00348

B. Dunn's Post-hoc Pairwise Test

Comparison	Z	P-unadjusted	P-adjusted (BH)
Rep200–Greengenes (Fresh Specimens)	-3.307	0.000943	0.00283
WoL–Greengenes (Fresh Specimens)	-2.058	0.0396	0.0594
Rep200 – WoL (Fresh Specimens)	1.196	0.232	0.232
Rep200–Greengenes (Museum Specimens)	-5.559	2.709e-08	8.126e-08
WoL–Greengenes (Museum Specimens)	-2.983	2.856e-03	4.284e-03
Rep200 – WoL (Museum Specimens)	2.630	8.549e-03	8.549e-03

Table 2A–B. Shannon's diversity index comparisons between the six reference processing types using Kruskal-Wallis rank sum test followed by Dunn's post-hoc pairwise testing (Benjamini-Hochberg corrected).

A. Kruskal-Wallis Rank Sum Test

Comparison	Kruskal-Wallis chi-squared	df	P-value
Museum Specimens	27.185	2	1.25e-06
Fresh Specimens	9.38	2	0.00919

B. Dunn's Post-hoc Pairwise Test

Comparison	Z	P-unadjusted	P-adjusted (BH)
Rep200 –Greengenes (Fresh Specimens)	-2.616	0.00889	0.0133
WoL–Greengenes (Fresh Specimens)	0.0707	0.944	0.944
Rep200 – WoL (Fresh Specimens)	2.687	0.00721	0.0216
Rep200–Greengenes (Museum Specimens)	-5.2118	1.871e-07	5.612e-07
WoL–Greengenes (Museum Specimens)	-2.735	6.240e-03	9.360e-03
Rep200 – WoL (Museum	2.477	1.325e-02	1.325e-02

Specimens)			

Figures

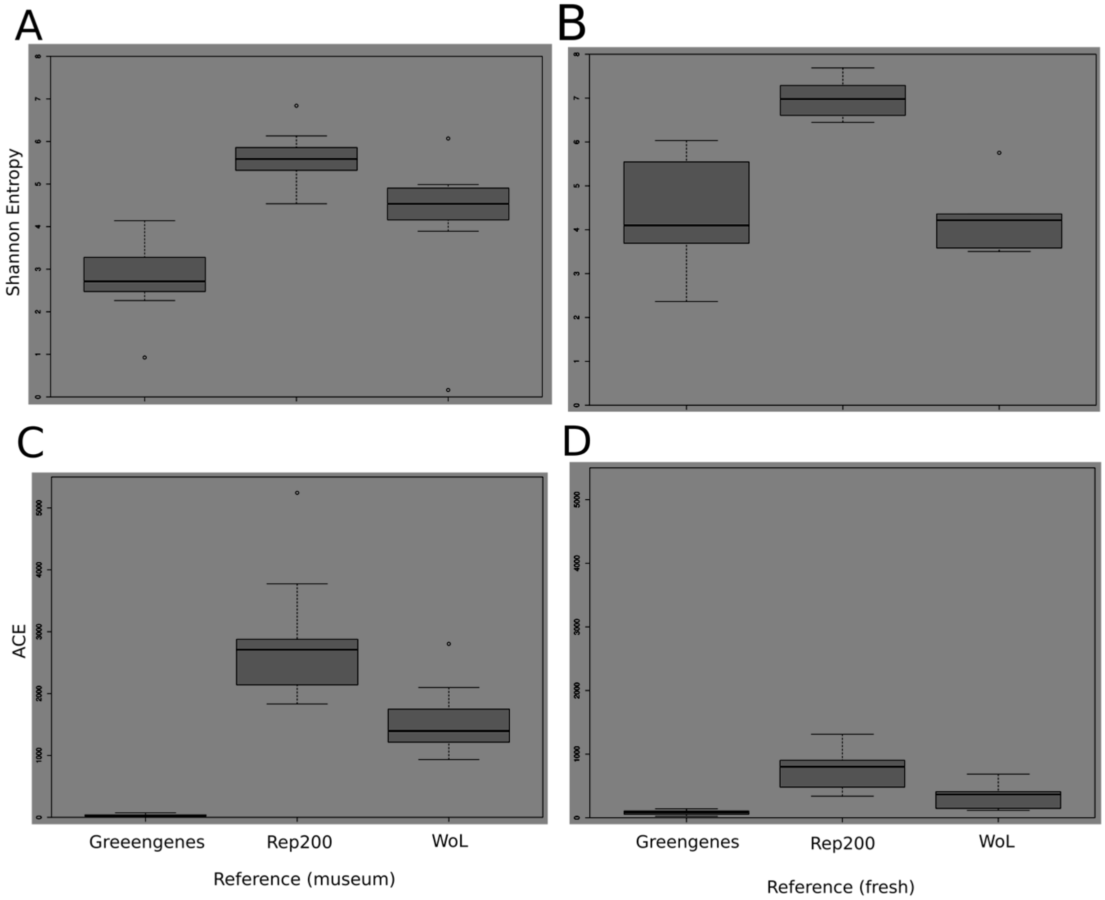

Figure 1 A–D. Boxplots of samples (n=18) of the shannon entropies for the different reference procedures from **(A)** museum specimens (n=13) and **(B)** fresh specimens (n=5) and the ACE values for the different reference procedures from **(C)** museum specimens (n=13) and **(D)** fresh specimens (n=5). The top and bottom of the boxes represent the upper and lower quartile boundaries, respectively. The black band is the median percentile. Bounds of the upper whisker is the default setting, calculated as min(max(x), Q_3 + 1.5 * IQR) and the bounds of the lower whisker is max(min(x), Q_1 – 1.5 * IQR).

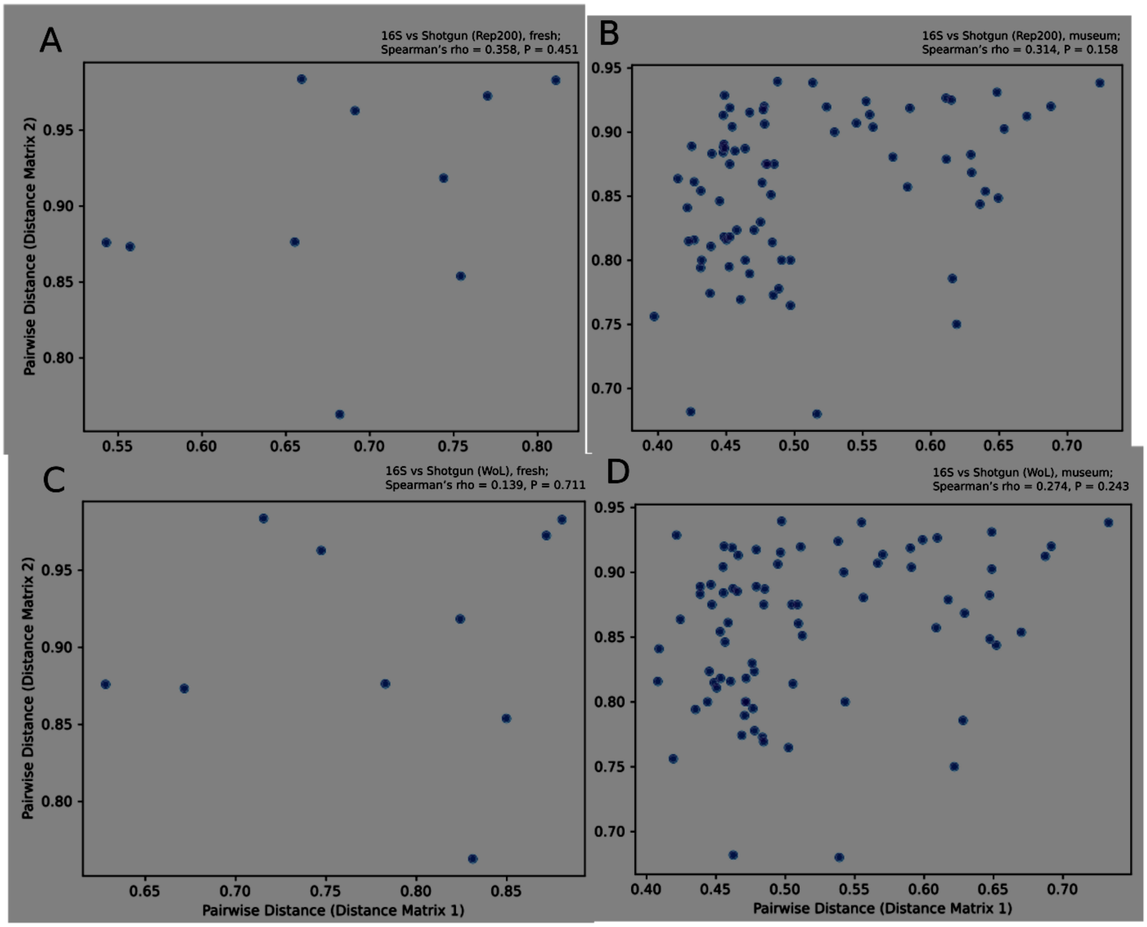

Figure 2 A–D. Mantel tests of Jaccard distance matrices from shotgun metagenomic sequencing data (Rep200 or WoL databases) and 16S rRNA gene sequencing data (Greengenes database). Fresh specimens (using Rep200) are compared in Panel A and museum specimens are compared in panel B. **(A)** Spearman's rho = 0.358, p-value = 0.451. **(B)** Spearman's rho = 0.314, P-value = 0.158. Fresh specimens (using WoL) are compared in Panel C and museum specimens are compared in panel D. **(C)** Spearman's rho = 0.139, p-value = 0.711. **(D)** Spearman's rho = 0.274, p-value = 0.243.

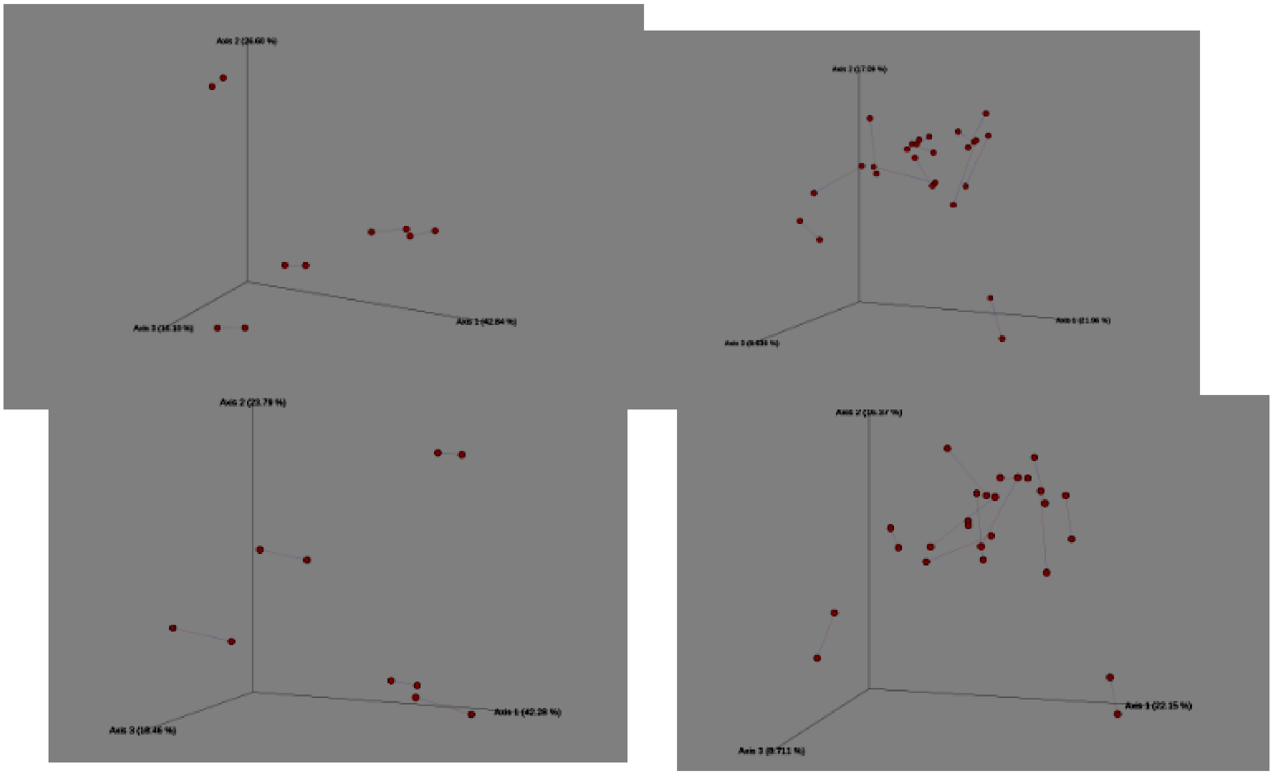

Figure 3 A–D. Procrustes analysis with Jaccard distance-matrix derived principal coordinates. The comparisons given are between: **(A)** Fresh specimen shotgun metagenomic sequencing (Rep200 database) and 16S rRNA gene sequencing (Greengenes database) with a true $M^2 = 0.0482$; p-value (of true M^2) = 0.219. **(B)** Museum-derived shotgun metagenomic sequencing (Rep200 database) and 16S rRNA gene sequencing (Greengenes database) with a true $M^2 = 0.481$; p-value (of true M^2) = 0.007. **(C)** Fresh specimen shotgun metagenomic sequencing (WoL database) and 16S rRNA gene sequencing (Greengenes database) with a true $M^2 = 0.0498$; p-value (of true M^2) = 0.475. **(D)** Museum-derived specimen shotgun metagenomic sequencing (WoL database) and 16S rRNA gene sequencing (Greengenes database). True $M^2 = 0.520$; p-value (of true M^2) = 0.019. A total of n=999 Monte Carlo simulations was used for all comparisons.

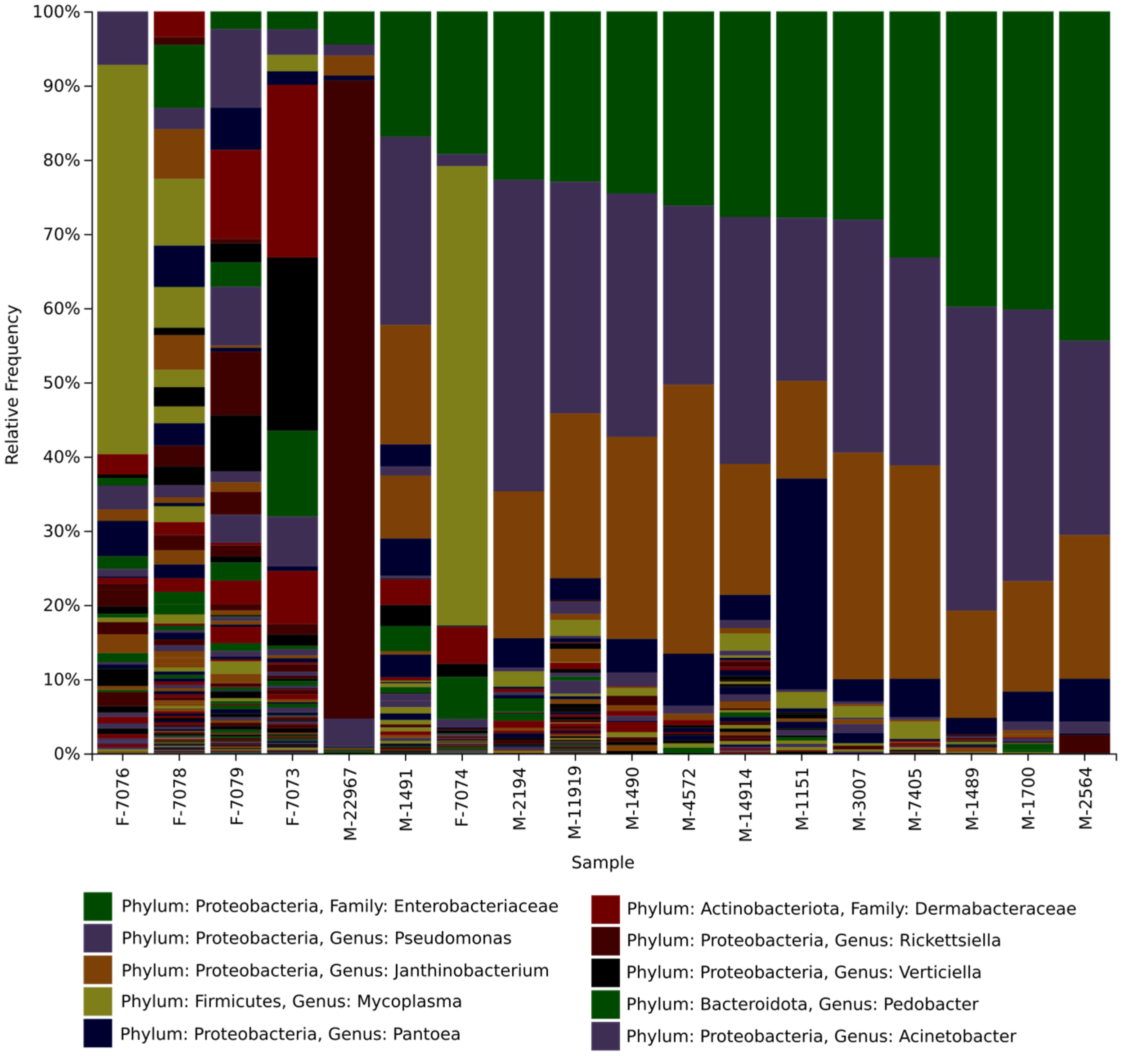

Figure 4. Abundance barplot of all specimens (n=18) with 16S rRNA gene sequencing derived taxonomies based on the Greengenes database. The top ten most commonly represented Genera are given in the legend. For Genus-level groupings not having a single known ID, the next highest known taxonomic level is given. Specimen ID is given for each column in addition to specimen type: F (fresh) or M (museum).

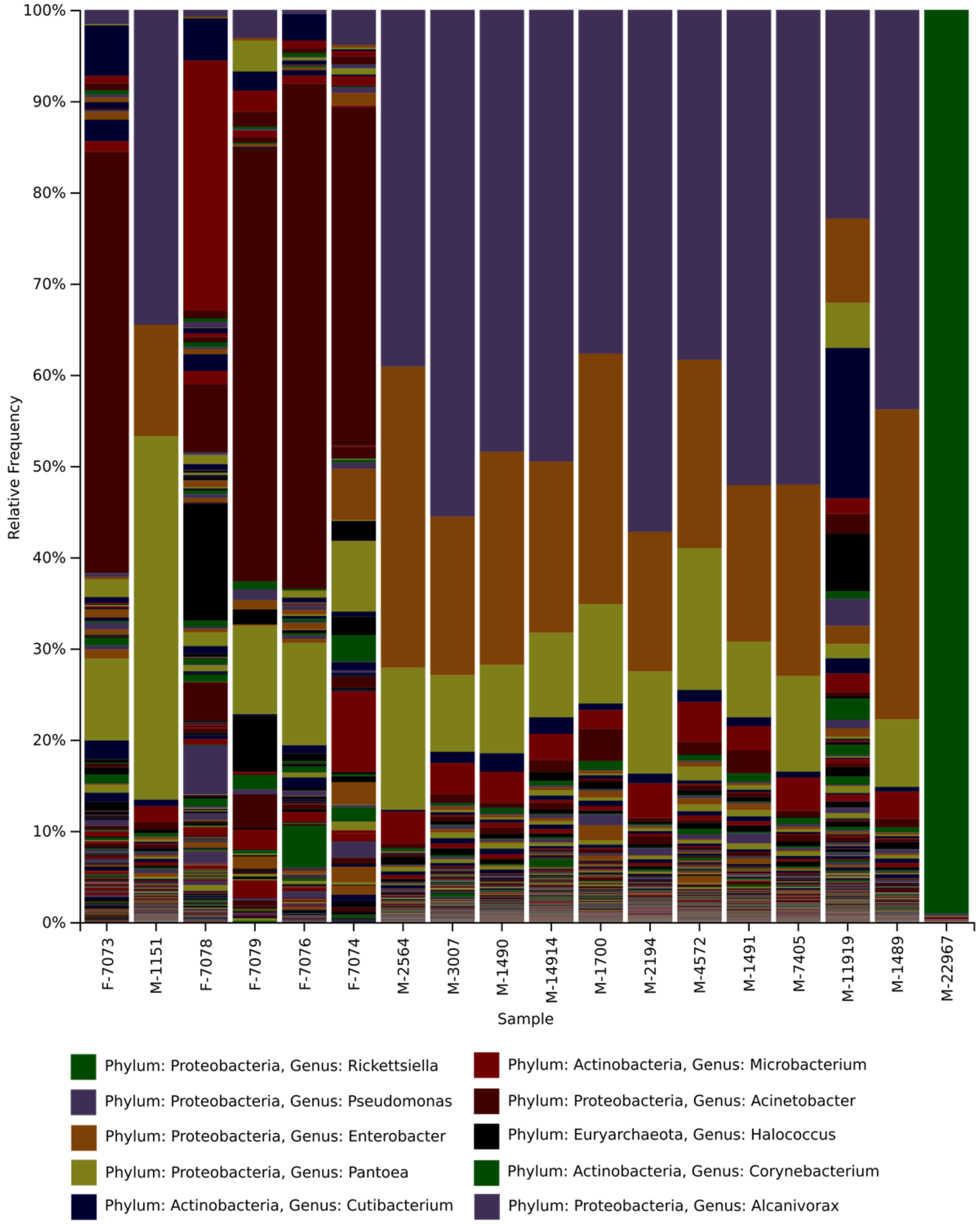

Figure 5. Abundance barplot of all specimen (n=18) shotgun metagenomic sequencing derived taxonomies based on the WoL database. The top ten most commonly represented Genera are given in the

legend. For Genus-level groupings not having a single known ID, the next highest known taxonomic level is given. Specimen ID is given for each column in addition to specimen type: F (fresh) or M (museum).

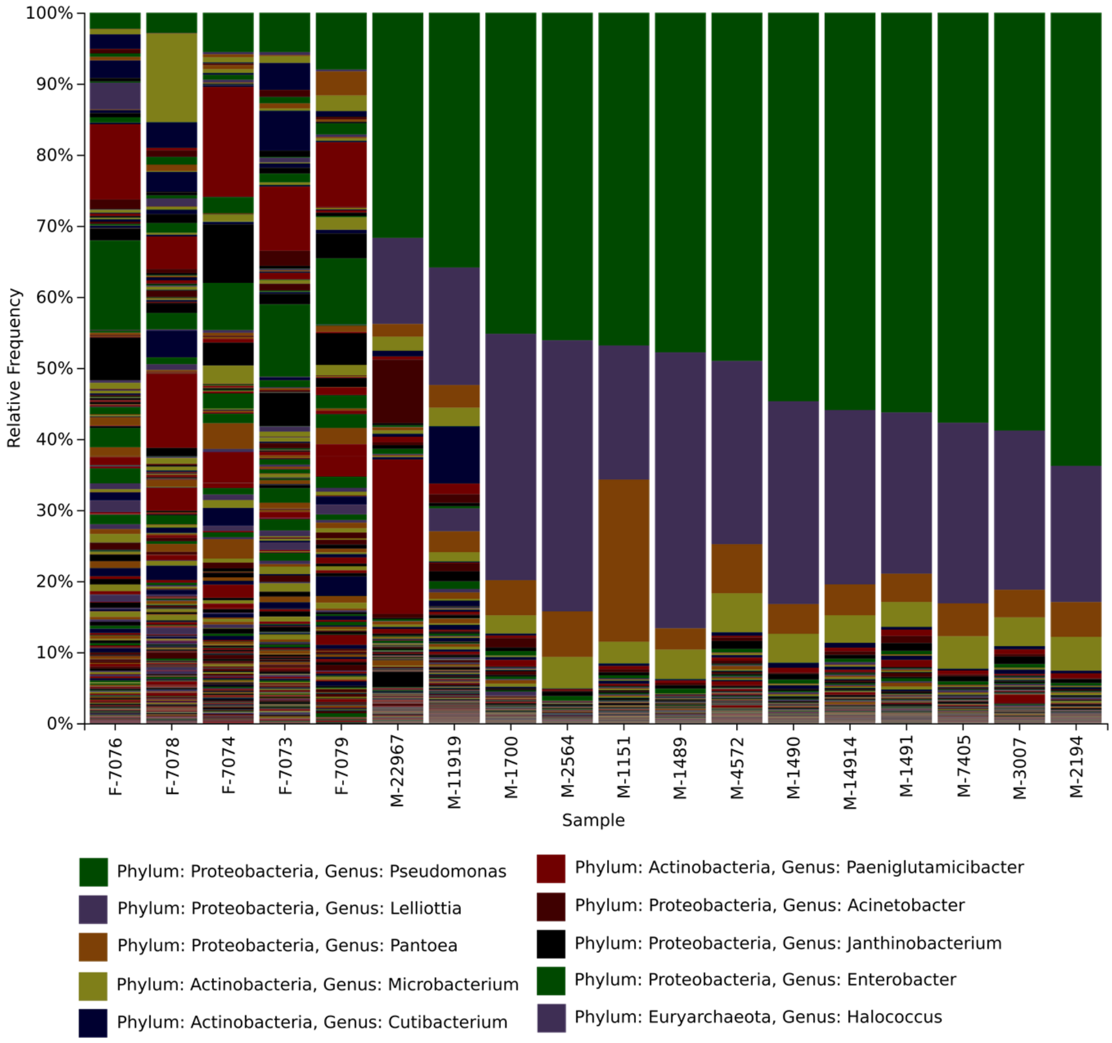

Figure 6. Abundance barplot of all specimen (n=18) shotgun metagenomic sequencing derived taxonomies based on the Rep200 database. The top ten most commonly represented Genera are given in the legend. For Genus-level groupings not having a single known ID, the next highest known taxonomic level is given. Specimen ID is given for each column in addition to specimen type: F (fresh) or M (museum).

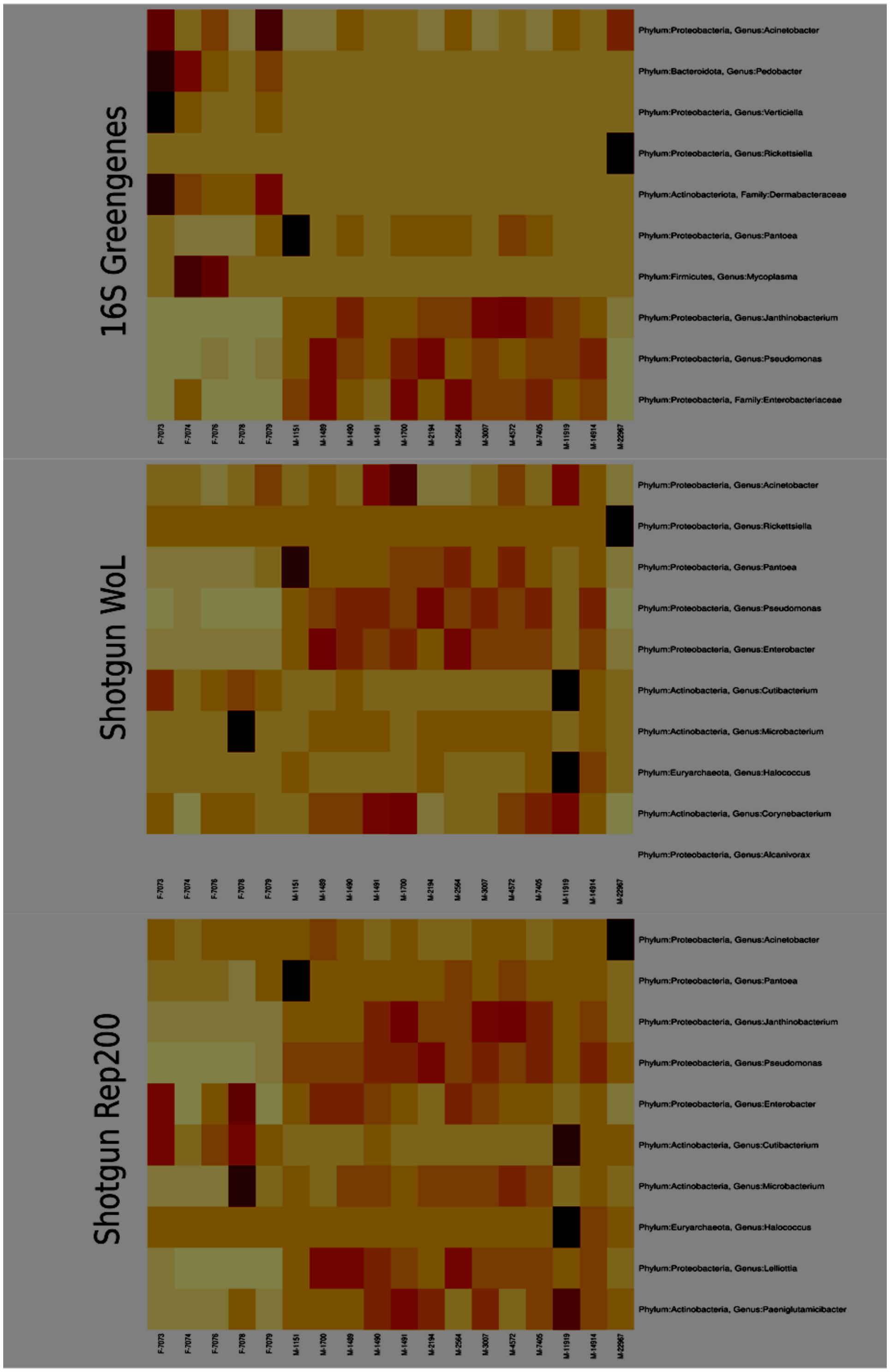
16S Greengenes
Phylum:Proteobacteria, Genus:Acinetobacter
Phylum:Bacteroidota, Genus:Pedobacter
Phylum:Proteobacteria, Genus:Verticiella
Phylum:Proteobacteria, Genus:Rickettsiella
Phylum:Actinobacteriota, Family:Dermabacteraceae
Phylum:Proteobacteria, Genus:Pantoea
Phylum:Firmicutes, Genus:Mycoplasma
Phylum:Proteobacteria, Genus:Janthinobacterium
Phylum:Proteobacteria, Genus:Pseudomonas
Phylum:Proteobacteria, Family:Enterobacteriaceae
F-7073
F-7074
F-7076
F-7078
F-7079
M-1151
M-1489
M-1490
M-1491
M-1700
M-2194
M-2564
M-3007
M-4572
M-7405
M-11919
M-14914
M-22967
Shotgun WoL
Phylum:Proteobacteria, Genus:Acinetobacter
Phylum:Proteobacteria, Genus:Rickettsiella
Phylum:Proteobacteria, Genus:Pantoea
Phylum:Proteobacteria, Genus:Pseudomonas
Phylum:Proteobacteria, Genus:Enterobacter
Phylum:Actinobacteria, Genus:Cutibacterium
Phylum:Actinobacteria, Genus:Microbacterium
Phylum:Euryarchaeota, Genus:Halococcus
Phylum:Actinobacteria, Genus:Corynebacterium
Phylum:Proteobacteria, Genus:Alcanivorax
F-7073
F-7074
F-7076
F-7078
F-7079
M-1151
M-1489
M-1490
M-1491
M-1700
M-2194
M-2564
M-3007
M-4572
M-7405
M-11919
M-14914
M-22967
Shotgun Rep200
Phylum:Proteobacteria, Genus:Acinetobacter
Phylum:Proteobacteria, Genus:Pantoea
Phylum:Proteobacteria, Genus:Janthinobacterium
Phylum:Proteobacteria, Genus:Pseudomonas
Phylum:Proteobacteria, Genus:Enterobacter
Phylum:Actinobacteria, Genus:Cutibacterium
Phylum:Actinobacteria, Genus:Microbacterium
Phylum:Euryarchaeota, Genus:Halococcus
Phylum:Proteobacteria, Genus:Lelliottia
Phylum:Actinobacteria, Genus:Paeniglutamicibacter
F-7073
F-7074
F-7076
F-7078
F-7079
M-1151
M-1700
M-1489
M-1490
M-1491
M-2194
M-2564
M-3007
M-4572
M-7405
M-11919
M-14914
M-22967

Figure 7. Heatmap of the ten (n=10) most highly represented Genera (of combined fresh and museum specimens) using 16S rRNA gene, Shotgun metagenomic (WoL), and Shotgun

metagenomic (Rep200) sequencing, respectively. Each row totals 100% (row normalized) with dark red approaching 100% and light yellow approaching 0%. For squares with <0.25%, 0 is assumed for purposes of visualization and normalization. For Genus-level groupings not having a single known ID, the next highest known taxonomic level is given. Specimen ID is given for each column in addition to specimen type: F (fresh) or M (museum).

Supplementary Results

This section contains supplementary figures and tables as referenced in the main manuscript.

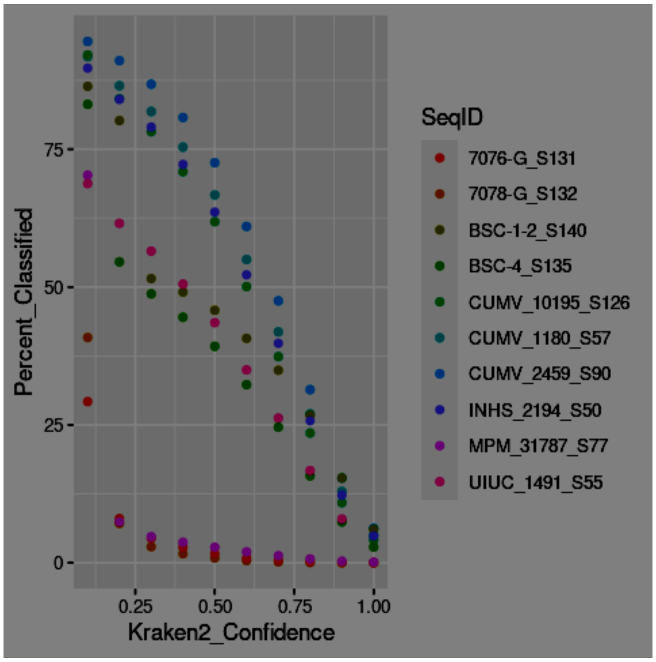

Supplementary Figure 1.
Examination of Kraken2 confidence threshold on reads classified for a subset of samples. These data indicate a qualitative leveling-off at 0.8, which was subsequently used as a conservative confidence threshold (high confidence) so as to avoid incorrect classification of real microbial sequences as background data.

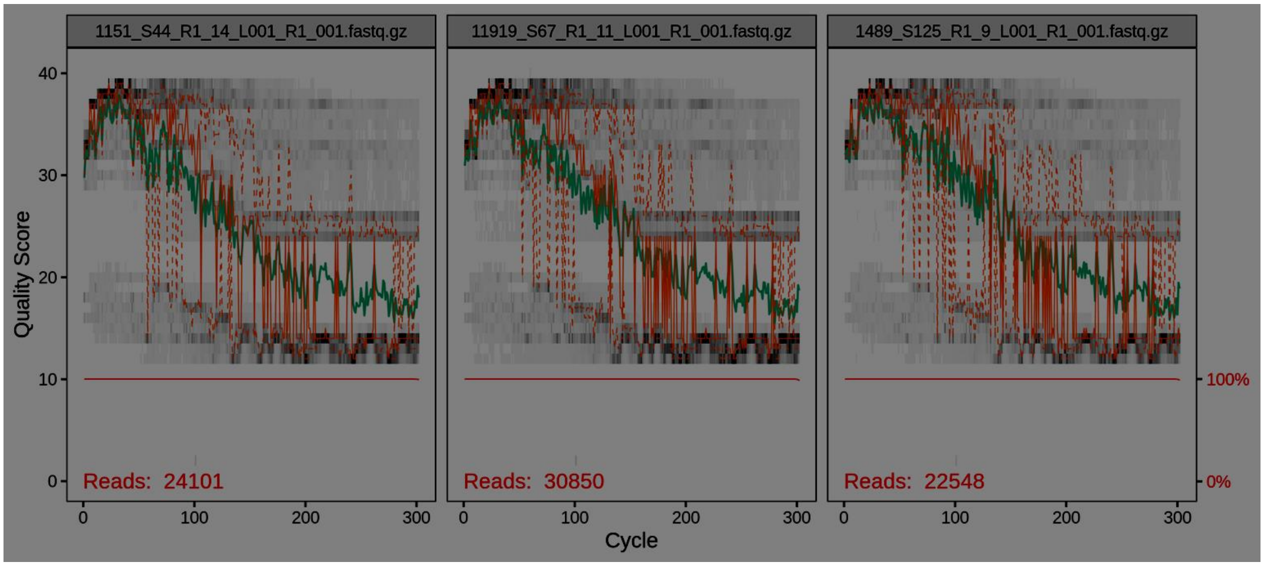
1151_S44_R1_14_L001_R1_001.fastq.gz
11919_S67_R1_11_L001_R1_001.fastq.gz
1489_S125_R1_9_L001_R1_001.fastq.gz
Quality Score
Reads: 24101
Reads: 30850
Reads: 22548
100%
0%
Cycle

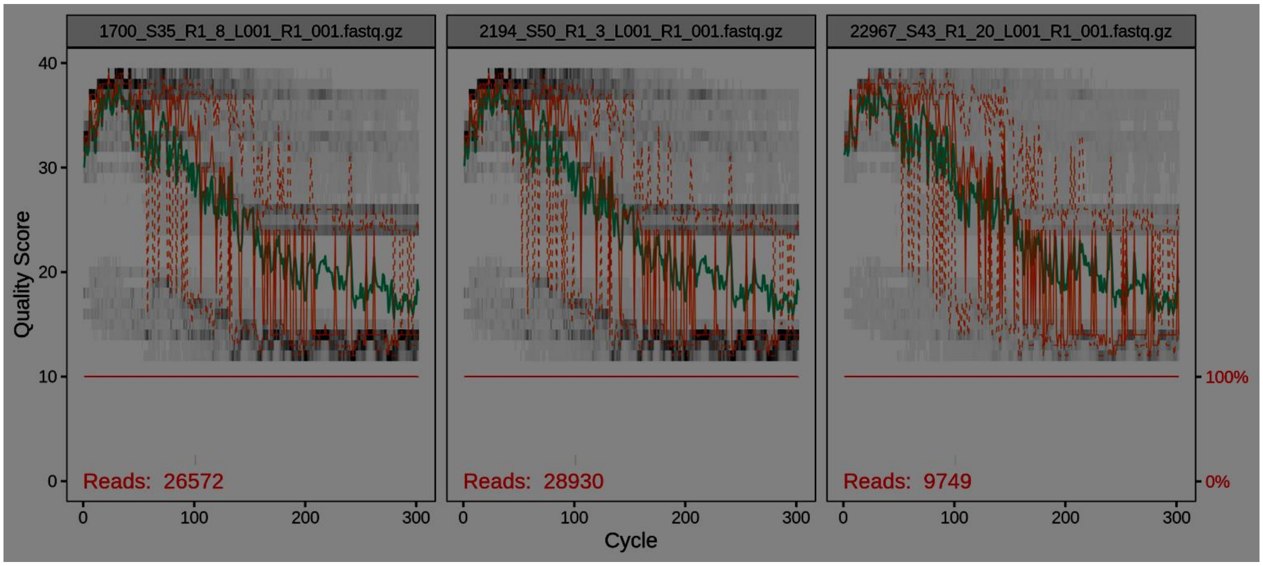
1700_S35_R1_8_L001_R1_001.fastq.gz
2194_S50_R1_3_L001_R1_001.fastq.gz
22967_S43_R1_20_L001_R1_001.fastq.gz
Quality Score
Reads: 26572
Reads: 28930
Reads: 9749
100%
0%
Cycle

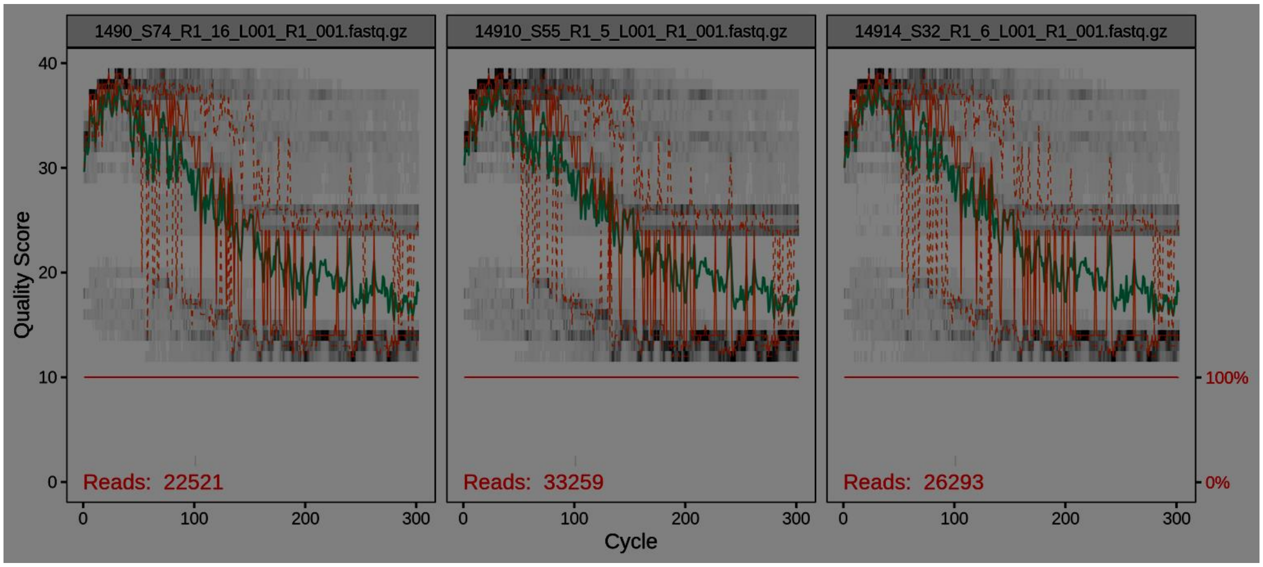
1490_S74_R1_16_L001_R1_001.fastq.gz
14910_S55_R1_5_L001_R1_001.fastq.gz
14914_S32_R1_6_L001_R1_001.fastq.gz
Quality Score
Reads: 22521
Reads: 33259
Reads: 26293
100%
0%
Cycle

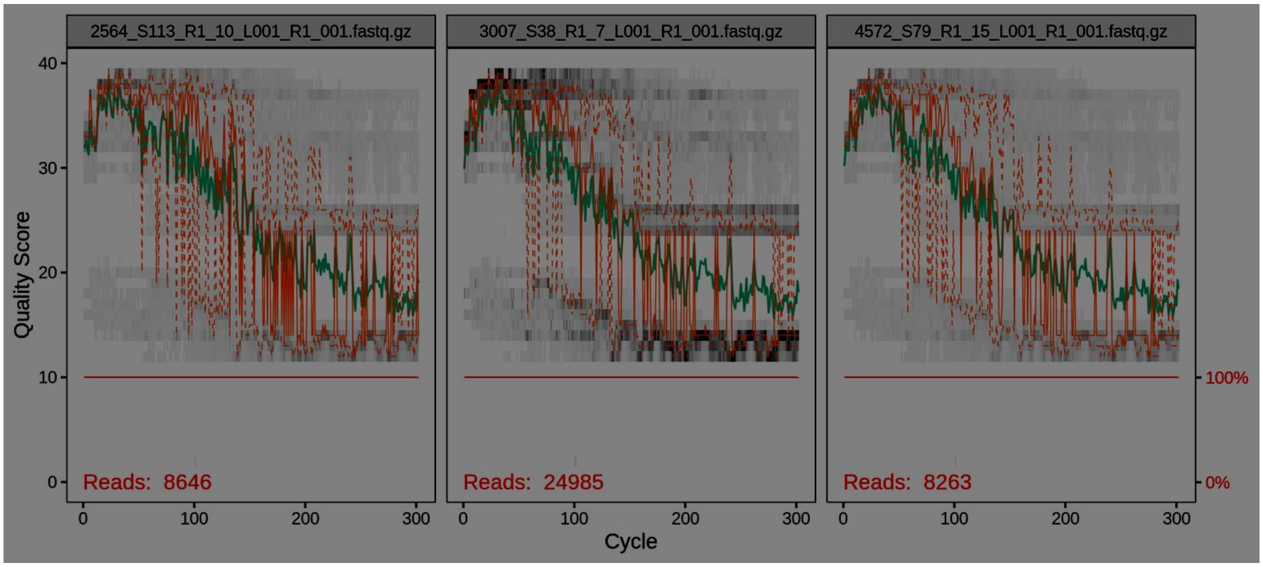
2564_S113_R1_10_L001_R1_001.fastq.gz
3007_S38_R1_7_L001_R1_001.fastq.gz
4572_S79_R1_15_L001_R1_001.fastq.gz
Quality Score
Reads: 8646
Reads: 24985
Reads: 8263
100%
0%
Cycle

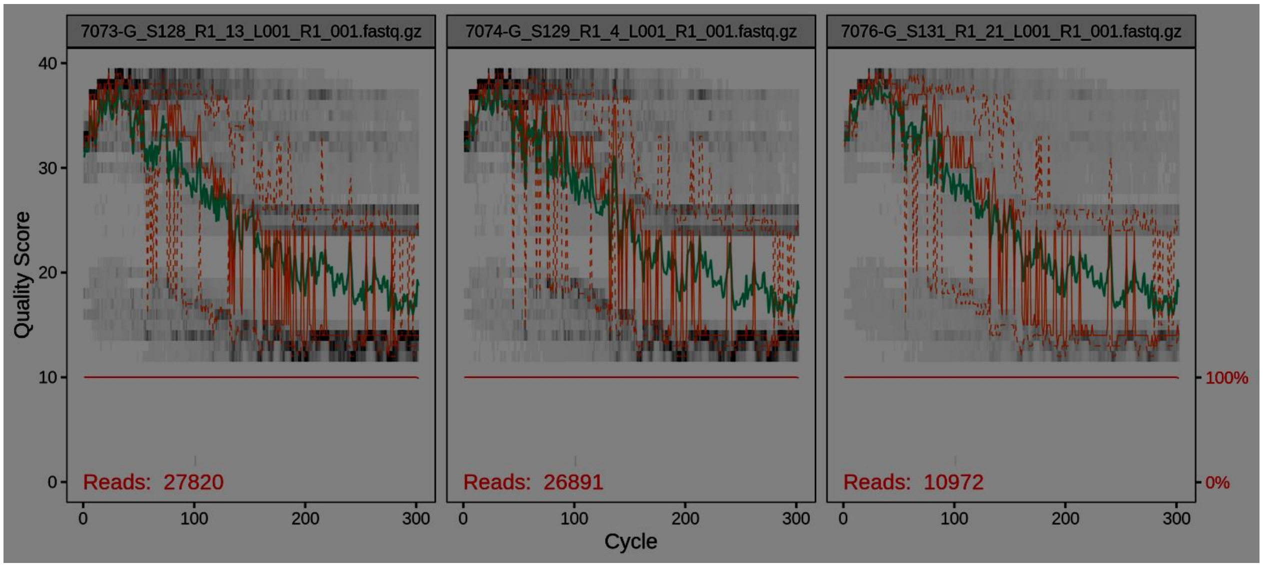

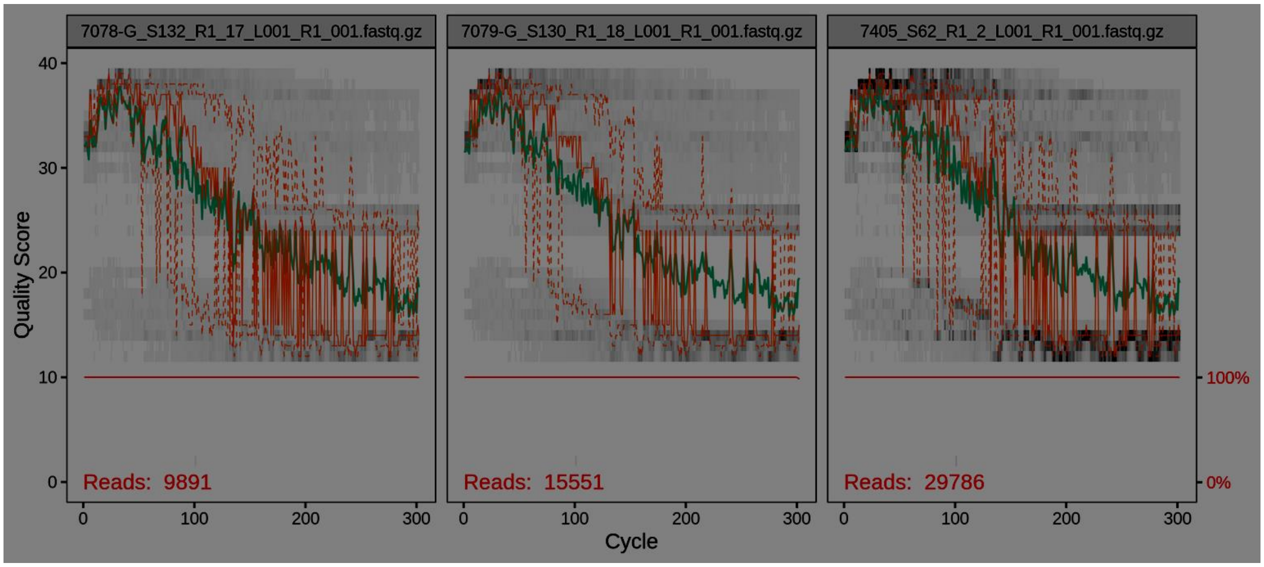

Supplementary Figure 2.
Panels A–R show the quality profile of reads corresponding to each sample analyzed from 16S rRNA gene sequencing. Quality score is given on the Y-axis and cycle number corresponding to read length is given on the X-axis. Reads were trimmed to 100bp based on these profiles, due to the quality decline for >100bp.

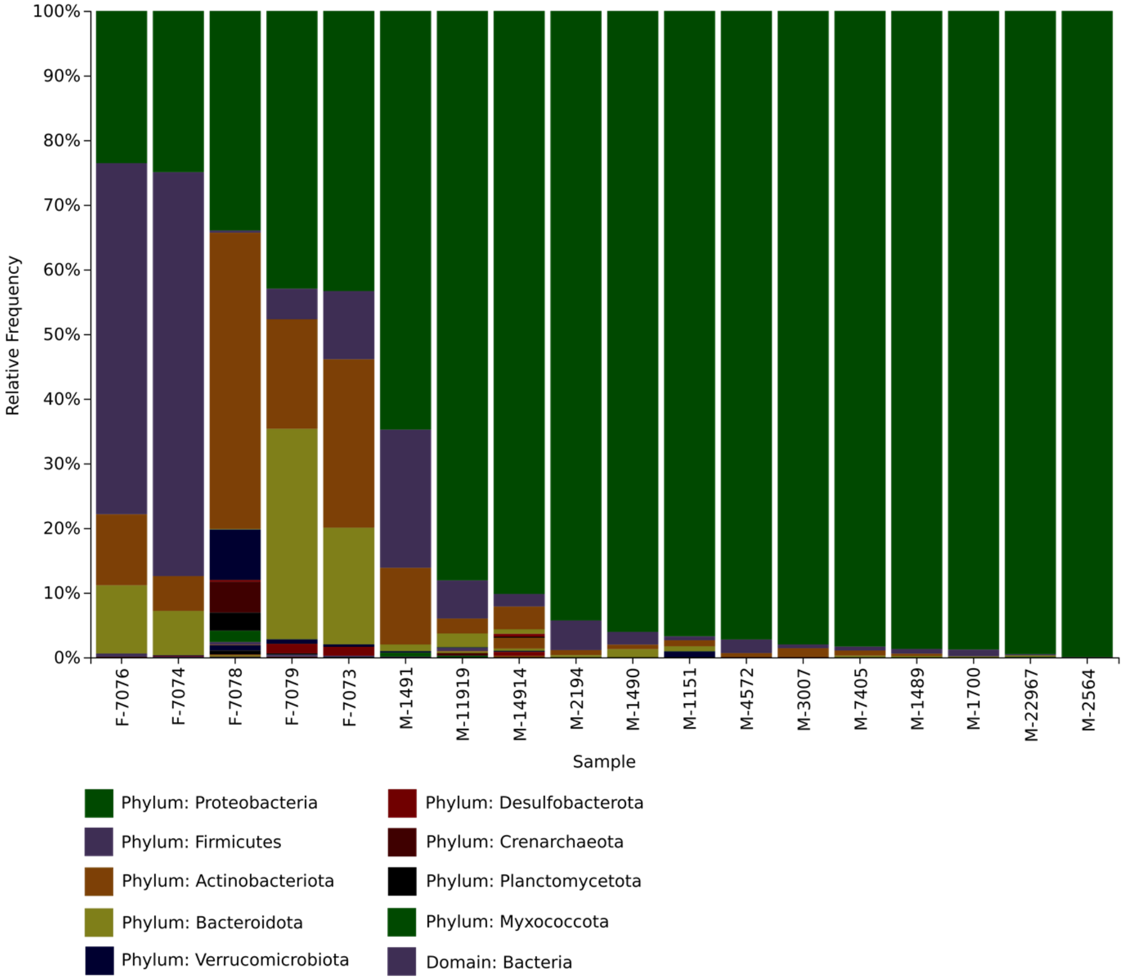

Supplementary Figure 3. Abundance barplot of all specimen (n=18) 16S rRNA gene sequencing derived taxonomies based on the Greengenes database. The top ten most commonly represented Phyla are given in the legend. For Phyla-level groupings not having a single known ID, the next highest known taxonomic level is given. Specimen ID is given for each column in addition to specimen type: F (fresh) or M (museum).

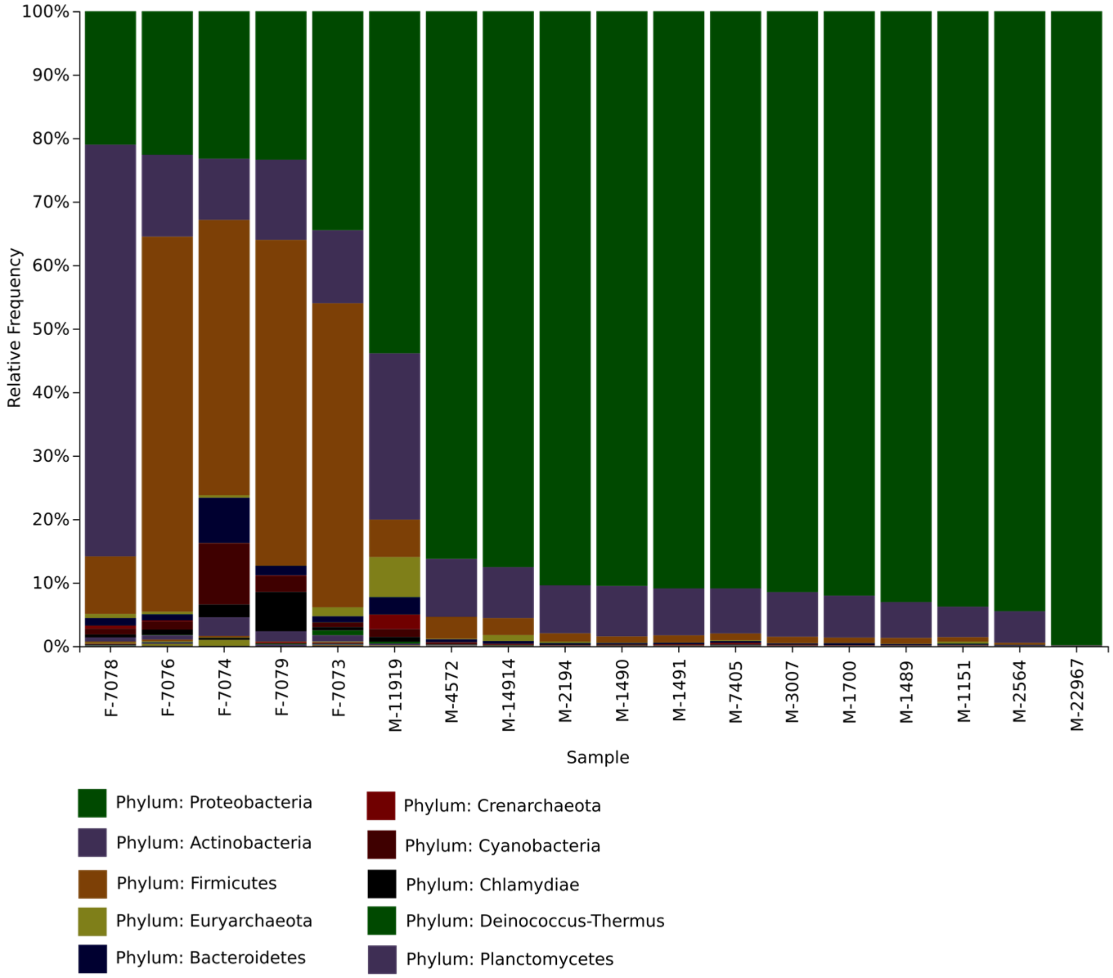

Supplementary Figure 4. Abundance barplot of all specimen (n=18) shotgun metagenomic sequencing derived taxonomies based on the WoL database. The top ten most commonly represented Phyla are given in the legend. For Phyla-level groupings not having a single known ID, the next highest known taxonomic level is given. Specimen ID is given for each column in addition to specimen type: F (fresh) or M (museum).

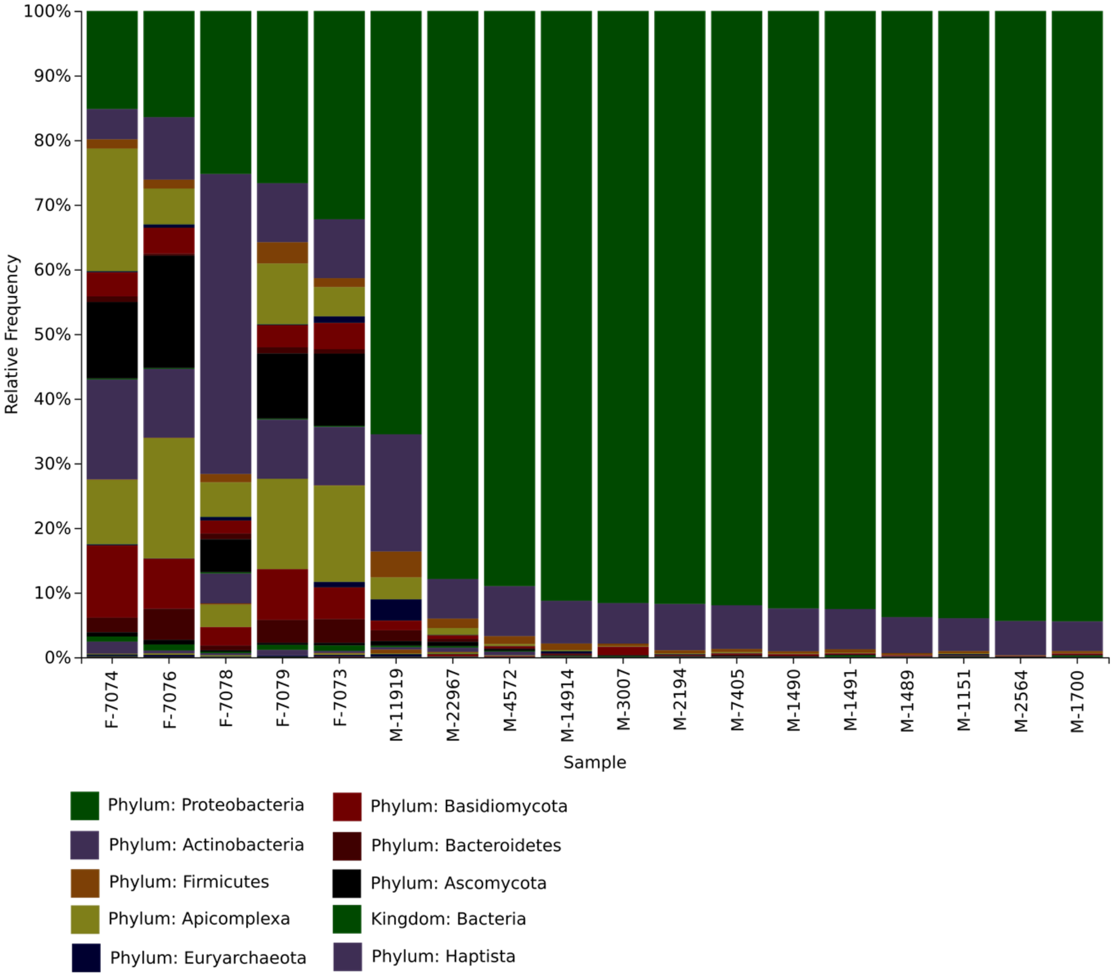

Supplementary Figure 5. Abundance barplot of all specimen (n=18) shotgun metagenomic sequencing derived taxonomies based on the Rep200 database. The top ten most commonly represented Phyla are given in the legend. For Phyla-level groupings not having a single known ID, the next highest known taxonomic level is given. Specimen ID is given for each column in addition to specimen type: F (fresh) or M (museum).

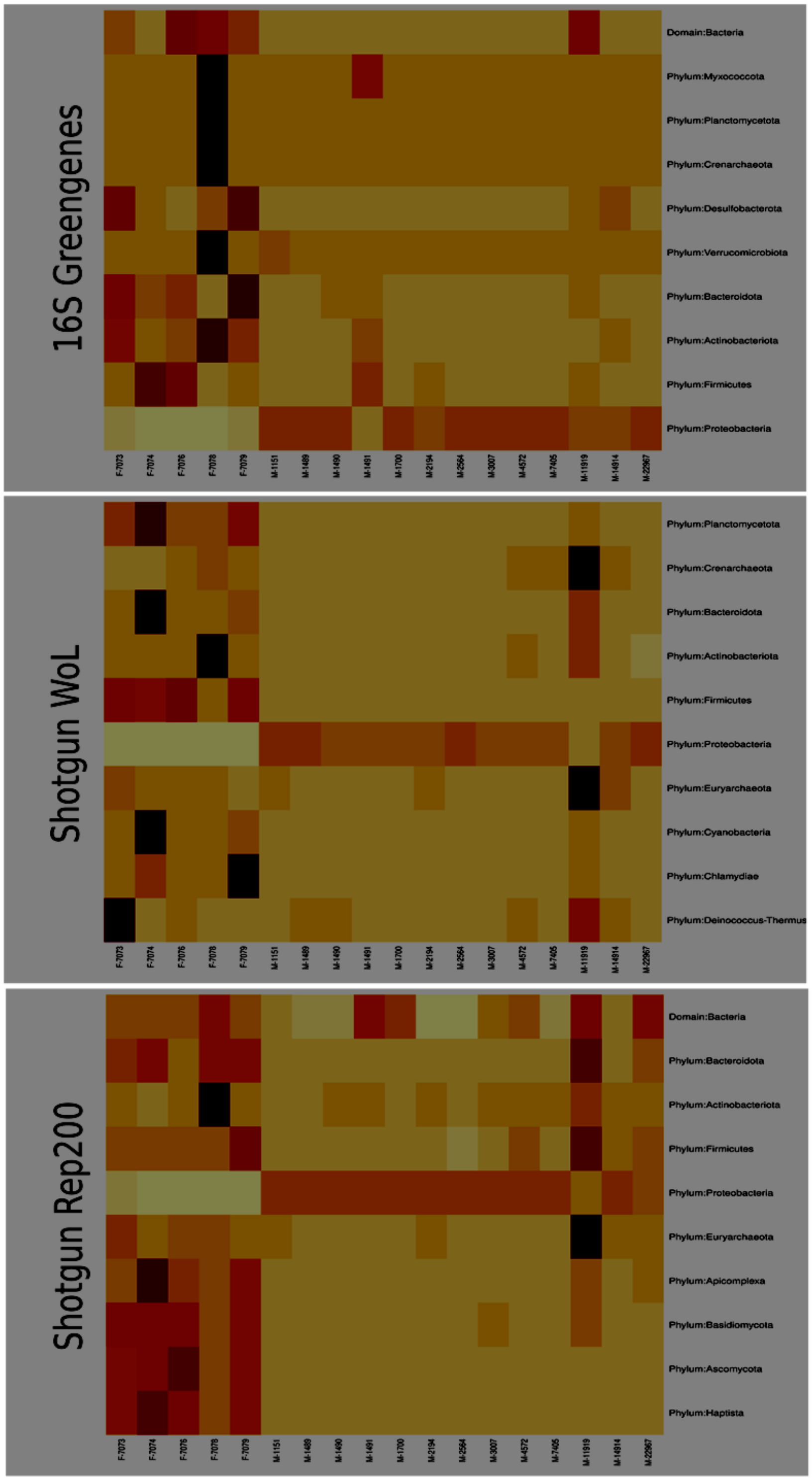
16S Greengenes
Domain:Bacteria
Phylum:Myxococcota
Phylum:Planctomycetota
Phylum:Crenarchaeota
Phylum:Desulfobacterota
Phylum:Verrucomicrobiota
Phylum:Bacteroidota
Phylum:Actinobacteriota
Phylum:Firmicutes
Phylum:Proteobacteria
Shotgun WoL
Phylum:Planctomycetota
Phylum:Crenarchaeota
Phylum:Bacteroidota
Phylum:Actinobacteriota
Phylum:Firmicutes
Phylum:Proteobacteria
Phylum:Euryarchaeota
Phylum:Cyanobacteria
Phylum:Chlamydiae
Phylum:Deinococcus-Thermus
Shotgun Rep200
Domain:Bacteria
Phylum:Bacteroidota
Phylum:Actinobacteriota
Phylum:Firmicutes
Phylum:Proteobacteria
Phylum:Euryarchaeota
Phylum:Apicomplexa
Phylum:Basidiomycota
Phylum:Ascomycota
Phylum:Haptista

Supplementary Figure 6. Heatmap of the ten (n=10) most highly represented Phyla (of combined fresh and museum specimens) using 16S rRNA gene, Shotgun metagenomic (WoL), and Shotgun metagenomic (Rep200) sequencing, respectively. Each row totals 100% (row normalized) with dark red approaching 100% and light yellow approaching 0%. For squares with <0.25%, 0 is assumed for purposes of visualization and normalization. For Phylum-level groupings not having a single known ID, the next highest known taxonomic level is given. Specimen ID is given for each column in addition to specimen type: F (fresh) or M (museum).

Supplementary Table 1. Shotgun metagenomic sequencing raw read (forward and reversed separated) counts and stats generated from 'seqfu count'.

**Sample/File**	**#Seq**	**Total bp**
1151_S44_R1_001	10074735	1521284985
11919_S67_R1_001	30165163	4554939613
1489_S125_R1_001	22345028	3374099228
1490_S74_R1_001	29571665	4465321415
14910_S55_R1_001	13760971	2077906621
14914_S32_R1_001	27332784	4127250384
1700_S35_R1_001	18119300	2736014300
2194_S50_R1_001	25643283	3872135733
22967_S43_R1_001	22190275	3350731525
2564_S113_R1_001	9108413	1375370363
3007_S38_R1_001	18791680	2837543680
4572_S79_R1_001	29748544	4492030144
7073-G_S128_R1_001	13087222	1976170522
7074-G_S129_R1_001	54875278	8286166978
7076-G_S131_R1_001	32746324	4944694924
7078-G_S132_R1_001	25236292	3810680092
7079-G_S130_R1_001	26314065	3973423815
7405_S62_R1_001	20082540	3032463540
1151_S44_R2_001	10074735	1521284985
11919_S67_R2_001	30165163	4554939613
1489_S125_R2_001	22345028	3374099228
1490_S74_R2_001	29571665	4465321415
14910_S55_R2_001	13760971	2077906621
14914_S32_R2_001	27332784	4127250384
1700_S35_R2_001	18119300	2736014300
2194_S50_R2_001	25643283	3872135733

22967_S43_R2_001	22190275	3350731525
2564_S113_R2_001	9108413	1375370363
3007_S38_R2_001	18791680	2837543680
4572_S79_R2_001	29748544	4492030144
7073-G_S128_R2_001	13087222	1976170522
7074-G_S129_R2_001	54875278	8286166978
7076-G_S131_R2_001	32746324	4944694924
7078-G_S132_R2_001	25236292	3810680092
7079-G_S130_R2_001	26314065	3973423815
7405_S62_R2_001	20082540	3032463540

Supplementary Table 2. Per sample read counts of the 16S rRNA gene sequencing data.

**Sample**	**Raw Counts (SE)**
1151	24101
11919	30850
1489	22548
1490	22521
14910	33259
14914	26293
1700	26572
2194	28930
22967	9749
2564	8646
3007	24985
4572	8263
7073	27820
7074	26891
7076	10972
7078	9891
7079	15551
7405	29786

Click here to access/download
Supporting Information
specimen_metadata_PLOS_ONE.csv
